# Palladium-Mediated Hydroamination of DNA-Conjugated Aryl Alkenes

**DOI:** 10.3389/fchem.2022.851674

**Published:** 2022-04-11

**Authors:** Kunliang Cai, Yuzhao Ran, Wenbo Sun, Sen Gao, Jin Li, Jinqiao Wan, Guansai Liu

**Affiliations:** HitGen Inc., Chengdu, China

**Keywords:** DNA-encoded library, palladium catalysis, C-N bond formation, DNA-conjugated aryl ethylene, hydroamination

## Abstract

C-N bond formation is one of the most commonly used reactions in medicinal chemistry. Herein, we report an efficient Pd-promoted hydroamination reaction between DNA-conjugated aryl alkenes and a wide scope of aliphatic amines. The described reactions are demonstrated in good to excellent conversions to furnish C (sp^3^)–N bonds on DNA. This DNA-compatible transformation has strong potentials for the application into DNA-encoded library synthesis.

## Introduction

DNA-encoded library technology (DELT), first conceptualized by Brenner and Lerner in 1992 Brenner and Lerner. (1992), has evolved into one of major platforms for small-molecule drug discovery. DNA-encoded libraries are built with combinatorial chemistries in a split-pool manner. Each individual compound in a DNA-encoded library is covalently attached to a unique DNA sequence, which serves as a barcode. The libraries, typically consisting of millions to billions of DNA-encoded molecules, can be pooled together and conveniently screened against biological targets with pharmaceutical interests in a single experiment. It has been recognized as a powerful and economic tool for hit identifications in both industry and academia (Gironda-Martínez et al., 2021).

One of the challenges in the area of DELT is to develop DNA-compatible chemistry for the synthesis of drug-like and diverse chemical entities as starting points for medicinal chemistry research (Zhu et al., 2019; Franzini and Randolph, 2016; Dickson and Kodadek, 2019; Götte et al., 2020). Most DNA-encoded library synthesis are reported to perform on unprotected DNA substrates (Shi et al., 2021; Fair et al., 2021); thus, the reaction conditions must be mild to preserve DNA information integrity, and consequently to remain its viability in PCR. In addition, chemical reactions used in DEL synthesis should have potentials to construct diverse encoded molecules by introducing multitudinous building blocks (BBs) through suitable functional groups on DNA (Zhang and Clark, 2021; Kalliokoski, 2015; Zabolotna et al., 2021), mainly represented by carboxylic acids, aldehydes, amines, aryl halides, etc.

**FIGURE 1:**
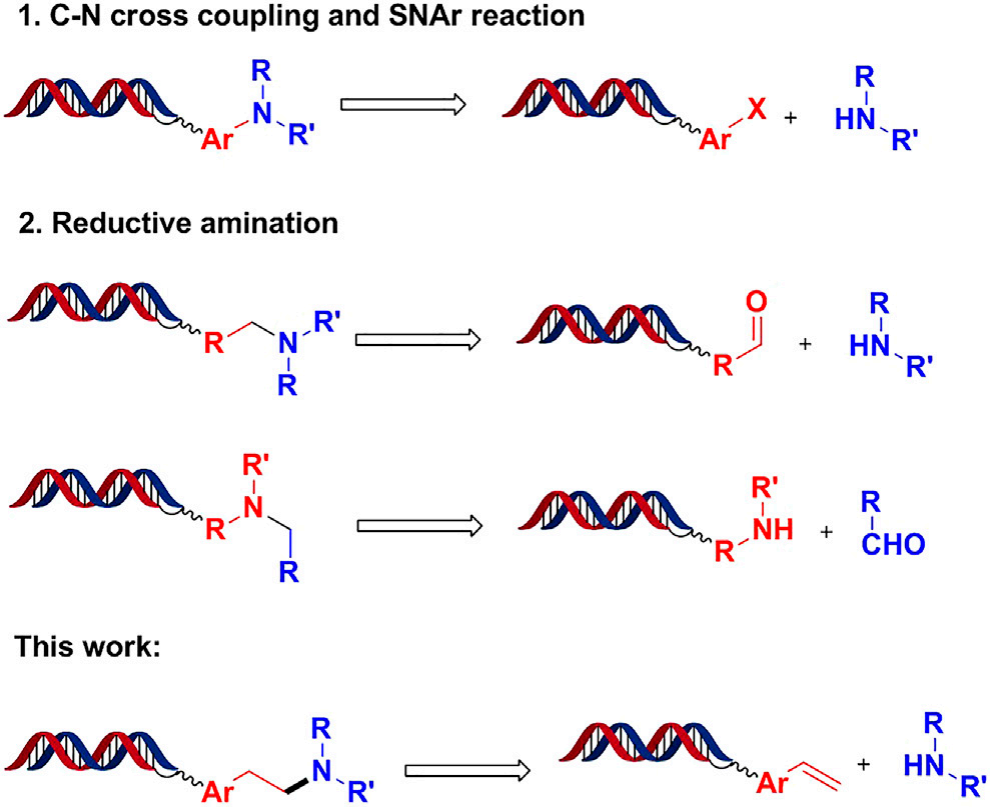
C-N bond formation for DEL synthesis.

C-N bond formation is of the most used chemistry transformations in medicinal chemistry due to the prevalence of nitrogen-containing motifs in approved drugs and bioactive compounds. C-N bond formation is extremely appealing for DEL synthesis because amines (or aldehydes) as BBs are easily accessible synthetic reagents (Zabolotna et al., 2021) for generating library diversity. Several on-DNA C-N bond formations, exemplified as C-N cross coupling (Buchwald coupling (Xu et al., 2019; de Pedro Beato et al., 2019; Chen et al., 2020; Lu et al., 2017), Ullmann coupling (Lu et al., 2017; Ruff and Berst, 2018), SNAr (Ding et al., 2018; Clark et al., 2009; Wang et al., 2019), and reductive amination (Flood et al., 2019; Ding et al., 2016), have been already reported for the synthesis of DELs. As depicted in Figure 1, these transformations require specific functional groups on DNA, such as DNA-tagged aryl halides, aldehydes, and amines. Herein, we describe the development of unprecedented Pd-promoted hydroamination reactions between DNA-conjugated aryl alkenes and a wide scope of aliphatic amines.

## Development and Results

### Condition Optimizations

The headpiece-tagged 4-vinylpyridine **1a** was used as a prototypical on-DNA substrate for the optimization of the hydroamination reactions in aqueous solutions (see headpiece structures in Supporting Information). After extensive reaction optimizations, the 2-(pyridin-4-yl)ethan-1-amine **3a** was obtained in 88% conversion (Table 1, entry 1). The reaction was dramatically promoted by the palladium catalyst. There was only trace amounts of **3a** detected under the condition without Pd(OAc)_2_ and most of the starting material **1a** was inert (entry 2). Other palladium catalysts, such as PdCl_2_ and Pd(PPh_3_)_4_, which are frequently employed in cross coupling reactions, led to diminished conversions (entries 3 and 4). We then presumed that the conversion was maximized by introducing large excesses of isopropyl amine **2a**. By comparison, 500 equivalents of **2a** in the standard condition gave better conversions than 200 equivalents in entry 5 and 100 equivalents in entry 6. This is in line with the kinetic mechanism for developing on-DNA intermolecular transformations. We finally found pH value of the final reaction solution played vital roles on conversions. As depicted in entry 7, the conversion was decreased to 55% without adding CsOH. In a reverse way, excess CsOH led to the final pH of the reaction solution to 12 that gave a diminished conversion (entry 8). Finally, we performed the reaction in 100 nmol scale under the optimal condition and found that it gave a comparable conversion as the small-scale reaction (Table 1, entry 9).

**TABLE 1:** Condition optimizations of C‐N bond formationa.

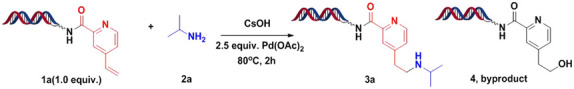				
**Entry**	**Deviation from standard condition**	**3a**	**4**	**1a**
1	none	88	<5	6
2	No Pd(OAc)_2_	6	<5	85
3	PdCl_2_	72	<5	20
4	Pd(PPh_3_)_4_	61	<5	27
5	200 equiv. **2a**, 0.4M in DMSO, 116mM	65	<5	28
6	100 equiv. **2a**, 0.2M in DMSO, 58mM	49	<5	42
7	no CsOH, pH∼9	55	<5	39
8	500 equiv. 5M in H_2_O, 290mM *p*H∼912	48	<5	11
9	100 nmol scale of **1a**	86	<5	8

^[a]^ Reaction conditions: DNA **1a** (10 nmol, 10 µL 0.25M borate buffer (pH=9.4)), **2a** (1 M in DMSO, 5 µL), CsOH (1 M in H_2_O, 1 µL); Pd catalyst (20 mM in DMSO, 1.25 µL), final pH∼10.5, DMSO/H_2_O=6.25/11, react under 80 °C for 2 hours. The conversion was determined by LC-MS.

### Scope Study of Amines

**SCHEME 1:**
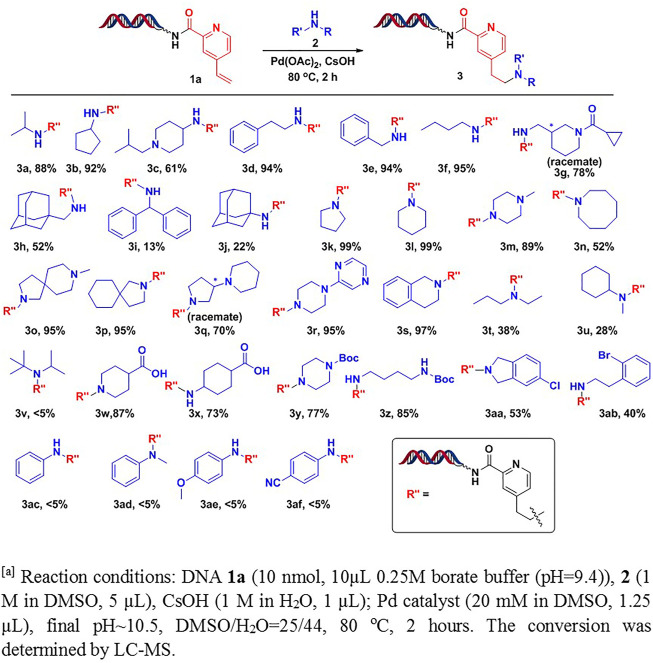
Scope study on amines Reaction conditions: DNA **1a** [10 nmol, 10 μL, 0.25 M borate buffer (pH = 9.4)], 2 (1 M in DMSO, 5 μL), CsOH (1 M in H_2_O, 1 μL); Pd catalyst (20 mM in DMSO, 1.2 μL), pH∼10.5, DMSO/H_2_O = 25/44, 80°C, 2 h. The conversion was determined by LC-MS.

With the optimal conditions in hand, we further studied substrate scopes of amines against headpiece-conjugated 4-vinylpyridine **1a** (Scheme 1). Under the conditions, we found that most primary alkyl amines were well tolerated, giving moderate to excellent conversions (**3a**-**3h**). The conversions were decreased when increasing steric hindrance of amines (**3i**-**3j**). Various types of secondary amines were also studied. The results showed that cyclic amines led to excellent conversions, such as monocyclic amines (**3k**-**3n**), spirocyclic amines (**3o**-**3p**), bicyclic amines (**3q**-**3r**), and fused cyclic amines (**3s**). However, non-cyclic secondary amines, especially with bulky groups (**3v**), resulted in low conversions (**3t**-**3v**). Interestingly, we found that carboxylic acids (**3w**-**3x**), Boc-protected diamines (**3y**-**3z**), and aryl halides (**3aa**-**3ab**), which were frequently used for diversity derivations, were well tolerated under the optimal conditions. The tolerance of these functional groups provided a straightforward pathway for further creating library diversities. Unfortunately, aromatic amines were not tolerated with these conditions. Only trace amounts of desired products were detected, with most starting material remained (**3ac**-**3af**).

### Cross Substrate Scope Study

**SCHEME 2:**
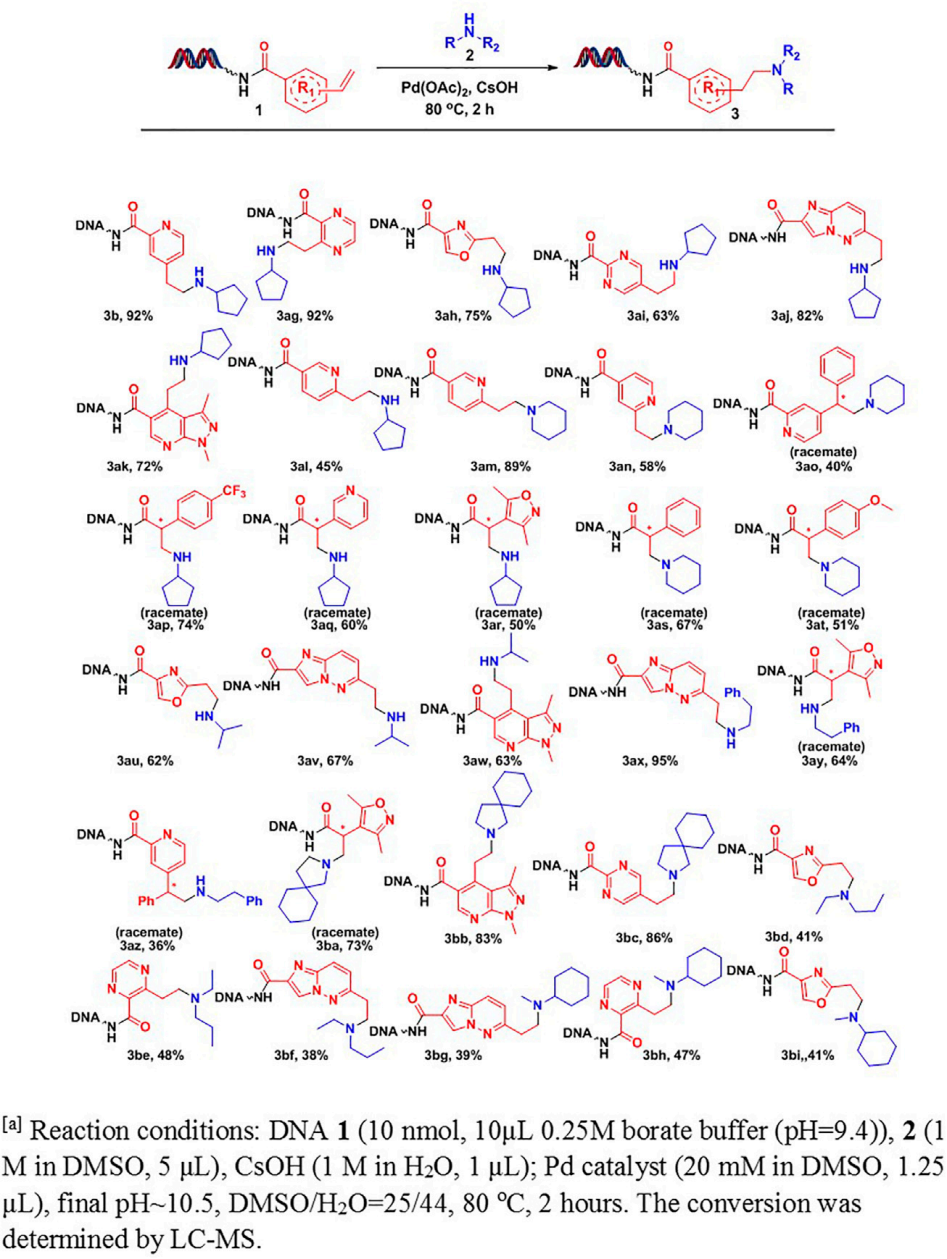
Scope study on DNA-conjugated aryl ethylene Reaction conditions: DNA 1 [10 nmol, 10 μL, 0.25 M borate buffer (pH = 9.4)], **2** (1 M in DMSO, 5 μL), CsOH (1 M in H_2_O, 1 μL); Pd catalyst (20 mM in DMSO, 1.2 μL), pH∼10.5, DMSO/H_2_O = 25/44, 80°C, 2 h. The conversion was determined by LC-MS.

Various DNA-conjugated alkenes (Scheme 2) and aliphatic amines were subjected to the optimal reaction condition for a comprehensive substrate scope study. As outlined in Scheme 2, we first investigated the effects of DNA-tagged alkenes on the conversions. Two types of on-DNA alkenes were prepared. We found both styrene analogs (**3b**-**3ao**) and acrylamide analogs (**3ap**-**3at**) delivered decent conversions to desired products. To our delight, except mono-substituted alkenes, many disubstituted alkenes (**3ao**-**3at**) also worked well under the condition. Furthermore, DNA-tagged alkenes containing heterocyclic moieties, such as pyridine (**3 aL**-**3am**, **3aq**), oxazole (**3ah**, **3au**, **3bd**, **3bi**), imidazo [1,2-b]pyridazine (**3aj**, **3av**, **3ax**, **3bf**, **3bg**), pyrazolo [3,4-b]pyridine (**3ak**, **3aw**, **3bb**), isoxazole (**3ar**, **3ay**, **3ba**), pyrimidine (**3ai**, **3bc**), and pyrazine (**3 ag**, **3be**, **3bh**), afforded the desired products in moderate-to-good conversions.

### Structural Confirmation

**SCHEME 3:**
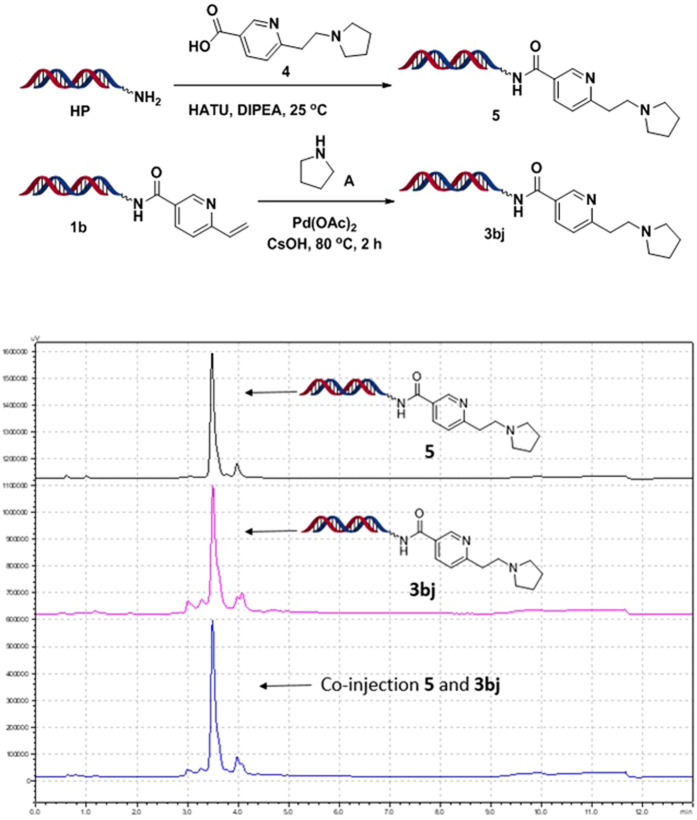
Co-injection experiments for identification.

To further confirm structures of the proposed products, co-injection experiments were carried out as shown in Scheme 3 (see details in Supporting Information). Small molecule **4**, which was structurally confirmed by ^1^HNMR, was conjugated with DNA *via* acylation reactions. The obtained conjugate **5** was compared with on-DNA synthesized conjugate **3bj** by co-injection experiments. Two samples gave identical m/z and HPLC trace, which indicated characterization of desired on-DNA product **3bj**. Besides, the off-DNA small-molecule synthesis with established on-DNA condition was conducted, and the results indicated that the desired structure was generated with good regioselectivity (see details in Supporting Information, Supplementary Figure S17–S18).

**SCHEME 4:**
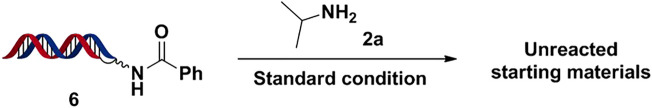
Control experiments for identification of reaction sites.

A control experiment was also performed under our optimal conditions by employing a benzoyl-substituted DNA **6** as a substrate (Scheme 4). The purpose of the experiment was to confirm that the conjugated small molecule rather than the DNA backbone underwent the reaction. As expected, quantitative recovery of starting material **6** was observed, thereby lending credence to our contention that the C-N bond formation did not take place on the DNA backbone.

### DNA Damage Test

To demonstrate the compatibility of this reaction with DNA chain and its suitability for subsequent DEL construction, the C-N bond formation reaction on long-chain DNA (headpiece-primer-code 1) followed by enzymatic ligation and quantitative PCR (qPCR) was evaluated (see detailed information in Supporting Information). The conversion on long-chain DNA with 33-bp dsDNA was found to be consistent with that on short-chain DNA under the same reaction condition. No evidence of DNA decomposition was detected by LC-MS or gel electrophoresis. Subsequent qPCR experiments indicated no obvious DNA damage on either case.

## Conclusion and Outlook

In conclusion, we developed an efficient method of an efficient palladium-mediated hydroamination reaction to construct C[sp3]–N bond between DNA-conjugated aryl ethylenes and amines. The methodology was demonstrated with moderate-to-excellent product conversions under mild conditions. It was verified in terms of no obvious damage to the DNA substrate, indicating the suitability of the DNA encoded library production.

## Data Availability

The original contributions presented in the study are included in the article/Supplementary Material, further inquiries can be directed to the corresponding authors.
