# Supplementary figures and images for: Palladium-Mediated Hydroamination of DNA-Conjugated Aryl Alkenes
Source: Front Chem. 2022 Apr 11;10:851674. doi: 10.3389/fchem.2022.851674 (PMC9035600; doi:10.3389/fchem.2022.851674)

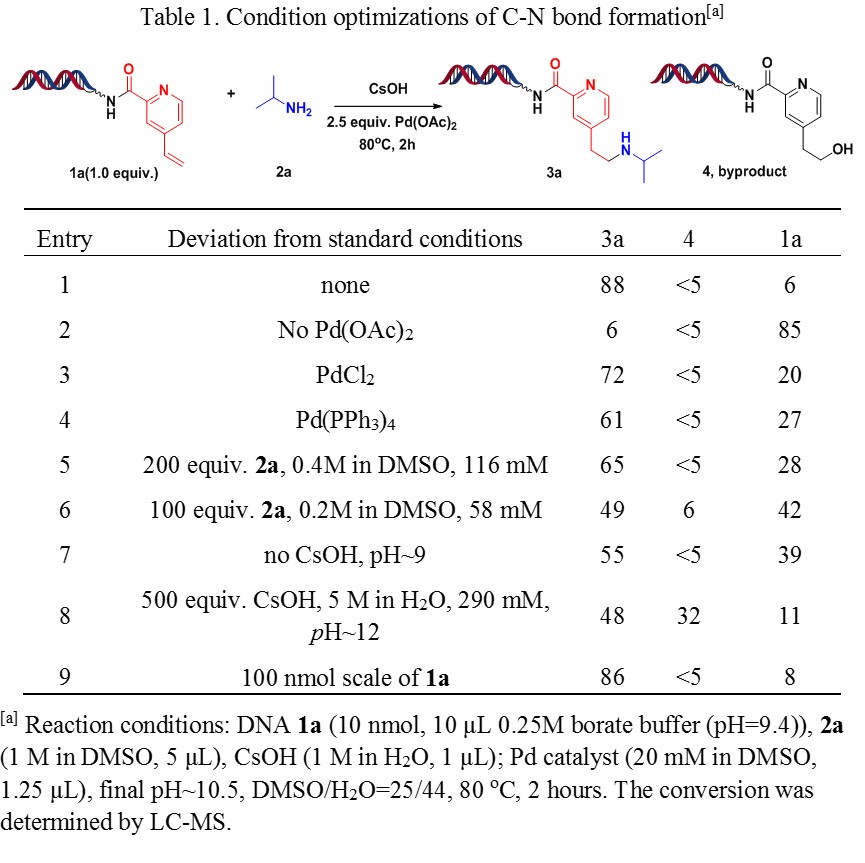

Supplement: Supplementary file 1 [file Image1.JPEG]
