# Supplementary material for: Palladium-Mediated Hydroamination of DNA-Conjugated Aryl Alkenes
Source: Front Chem. 2022 Apr 11;10:851674. doi: 10.3389/fchem.2022.851674 (PMC9035600; doi:10.3389/fchem.2022.851674)
Supplement: Supplementary file 2 [file DataSheet1.docx]

Supplementary Material

1. General information

**Materials**

The chemically modified DNA oligonucleotide headpiece (HP, 5’-/5Phos/GAGTCA/iSp9/iUniAmM/iSp9/TGACTCCC-3’, Figure S1) was synthesized at HitGen Inc. T4 ligase was expressed at HitGen Inc. and its activity was determined through testing DNA oligomer ligations on HP.

All buffer and ionic solutions, including ligation buffer, aq. NaOH (1 M), aq. NaOH ( 200 mM), aq. NaCl (5 M), aq. HCl (1 M), basic borate buffer (250 mM sodium borate/boric acid, pH 9.4), phosphate buffer (250 mM sodium dihydrogen phosphate/disodium hydrogen phosphate, pH 5.5), acetate buffer (500 mM sodium acetate/acetic acid, pH 7.1), acetate buffer (500 mM sodium acetate/acetic acid, pH 8.1) were prepared in-house.

The ligation buffer stock used in ligation reactions was composed as follows: 500 mM Tris pH 7.5, 500 mM NaCl, 100 mM MgCl_2_, 100 mM DTT and 25 mM ATP.

Chemical building block and reagents were sourced from a variety of vendors, and were generally used from aliquots dissolved in DMSO, DMA, MeCN or THF depending on solubility and optimized reaction conditions.

**Figure S1.** Headpiece (5’-/5Phos/GAGTCA/iSp9/iUniAmM/iSp9/TGACTCCC-3’), MW = 4937.2

**General procedure for the analysis of oligonucleotide compositions**

DNA was characterized on UPLC-MS system equipped with PDA and QDa mass detector (Waters, MA, USA). The UPLC system was set as following. Column: Acquity UPLC Oligonucleotide BEH C18 Column, 130A, 1.7 μm, 2.1 mm × 50 mm, and maintained at 40 ^o^C. Mobile phases: 0.75 % HFIP/0.0375 % DIPEA/10 μM EDTA in HPLC grade water (A) and 0.75 % HFIP/0.0375 % DIPEA/10 μM EDTA in 80/20 HPLC grade methanol/water (B). Eluting gradient: from 26 % to 46 % of B in 1.2 minutes, flow rate 0.3 ml/min. Absorption was detected at 260 nm. Electrospray ionization (ESI) probe temperature was 600 ^o^C, source temperature was 120 ^o^C and ESI capillary was 0.8 kV. Mass detector (QDa) was operated at negative full scan mode in the range of 500-1200 (m/z). Data was analyzed by ProMass HR 2.0 (Novatia, Pennsylvania, USA) and MassLynx4.1 (Waters, MA, USA).

**General method for conversion calculation of DNA tagged material through LCMS**

The conversions for DNA-encoded chemistries were determined by intensity calculation in TIC trace.


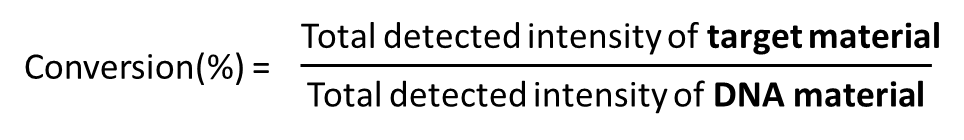


Example for conversion calculation of **3m** determined by LCMS:


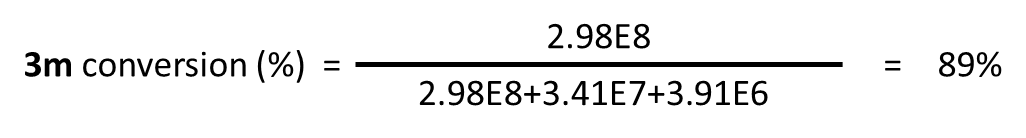


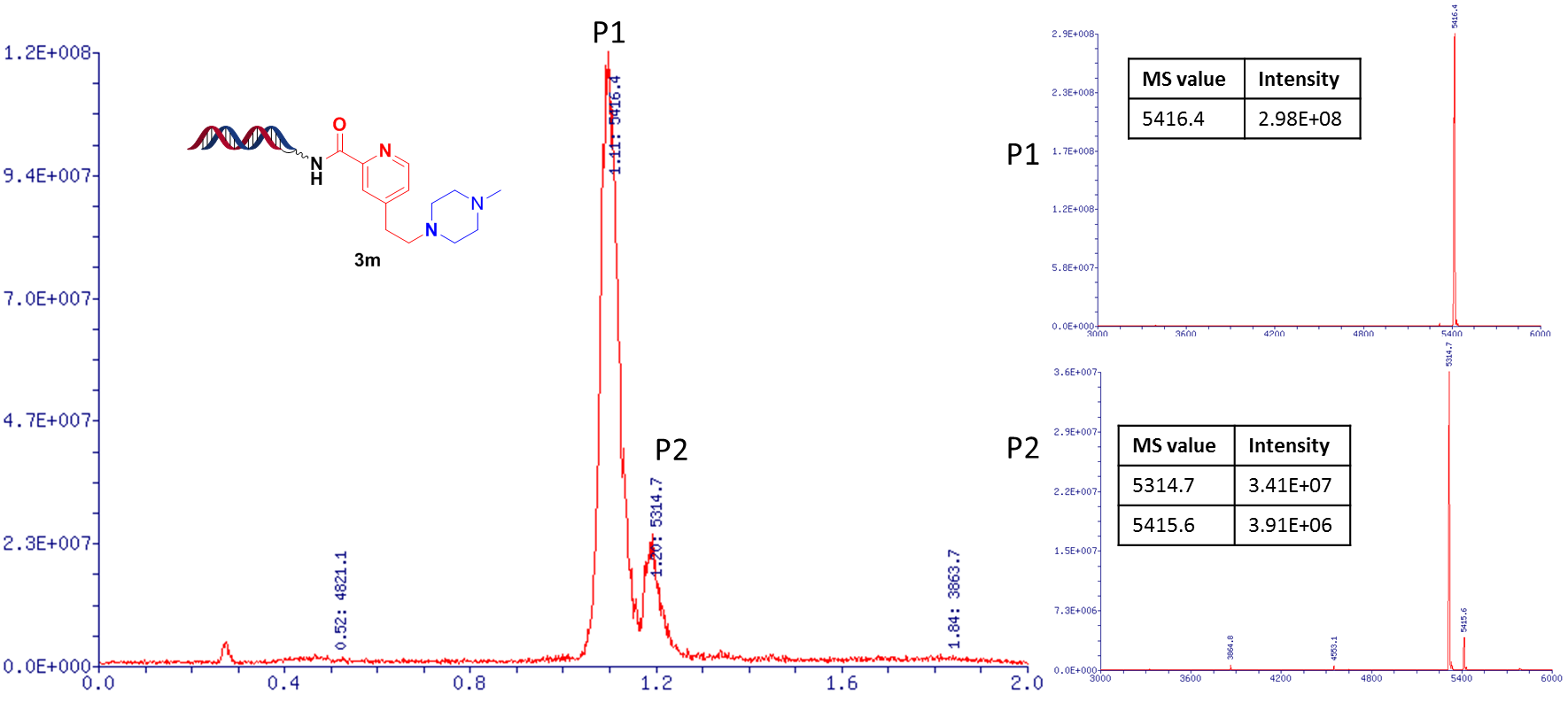


**General procedure for ethanol precipitation**

To a DNA reaction mixture was added 10% (V/V) 5 M NaCl solution and 3 times the volume of absolute ethanol. The solution was then incubated in dry ice for 2 h. The precipitated material was then isolated as a pellet by centrifugation and subsequent removal of the supernatant. 75% aq. ethanol was then added to the pellet and the mixture was centrifuged again. The supernatant again was discarded and the DNA pellet was dried in air or under gentle vacuum.

**General procedure for DNA ligation (HP and primer 1 ligation as an example)**

This reaction contained variably derivatized HP starting material (500 nmol in H_2_O, 1 equiv), P1 (833 nmol in H_2_O, 1.67 equiv), 10x ligation buffer (333 μL), T4 DNA ligase (47.9 μL, 14.0 μg/μL) and nuclease-free water (to total volume of 3332 μL). The reaction mixture was incubated at 20 ^o^C overnight before performing gel analysis. The crude material was purified by ethanol precipitation and taken on to next step synthesis without further purification.

Ligation reactions were monitored by gel electrophoresis on 3 % agarose gel in TBE buffer (40 mM Tris-Cl, 45 mM boric acid, 1 mM EDTA, pH 8.3) system referenced by a 50 bp DNA ladder (Takara, Japan). Before gel loading, the DNA samples were mixed with 0.20 volumes of the 6x gel-loading buffer (Takara, Japan) containing 0.2 % GelRed nucleic acid gel stain (Biotium, USA). Then, 30 pmol of treated DNA samples were loaded on gel and the gel was run at 135 V for 35-40 min. DNA fragments were visualized and analyzed by Bio-Rad ChemiDoc XRS+ Imaging system (Bio-Rad, CA, USA).

**General procedures for on-DNA substrate preparation**

**Elaboration of HP to HP-AOP-NH_2_ for substrate preparation.** All substrates were prepared on HP that had been further elaborated by a long amino-terminating linker. This elaborated HP, HP-AOP-NH_2_, was prepared through amidation of 1-(9H-fluoren-9-yl)-3-oxo-2,7,10,13,16-pentaoxa-4-azanonadecan-19-oic acid through the general acylation procedure and Fmoc deprotection.

**The preparation of Fmoc protected HP**

In a 15 mL Falcon tube, HP (2 μmol) was dissolved in sodium borate buffer (250 mM, pH 9.4, 2 mL). The stock solutions of 1-(9H-fluoren-9-yl)-3-oxo-2,7,10,13,16-pentaoxa-4-azanonadecan-19-oic acid (10 equiv, 100 μL, 200 mM in DMA), HATU (10 equiv, 50 μL, 400 mM in DMA), and DIPEA (10 equiv, 50 μL, 400 mM in DMA) were first chilled at 0 °C for 5 minutes and then mixed. The mixed reagents were further chilled at 4 °C for 5 minutes and finally added to HP solution in sodium borate buffer. The reaction was proceeded at room temperature for 30 minutes. The product was obtained by ethanol precipitation as described above. The DNA pellet was re-dissolved in 2 mL dd-H_2_O and used directly without further purification (98 % yield). Calculated MS 5406.7, found 5407.2, observed m/z (product) =900.2 [M-6H]^6-^

**Figure S2.** Fmoc protected HP (MW = 5406.72)

**The preparation of HP-AOP-NH_2_**

In a 15 mL Falcon tube (Corning), 200 μL piperidine was added to a solution of Fmoc protected HP (2 μmoL in 2 mL dd-H_2_O, Figure S2). The reaction was proceeded at room temperature for 30 minutes. The product was obtained by ethanol precipitation as described above (95 % yield). Calculated MS 5184.4, found 5184.8, observed m/z (product) = 647.1 [M-8H]^8-^

(Abbreviated as: )

**Figure S3.** HP-AOP-NH2 (MW = 5184.4)

**On-DNA substrates were obtained by treating headpiece and small molecules with the following protocols**

**Amide formation (amines on DNA):** HP-AOP-NH_2_ was dissolved in sodium borate buffer (250 mM, pH 9.4) to make 1 mM solution. Acid (200 mM in DMA, 50 equiv), HATU (200 mM in DMA, 50equiv), and DIPEA (200 mM in DMA, 50 equiv) were firstly chilled at 4 ^o^C for 5 minutes, and then mixed together. The mixture was chilled at 4 ^o^C for 5 minutes, then transferred to HP-AOP-NH_2_ solution. The reaction was allowed to proceed at room temperature for 2 hrs.

1. Experimental procedures

**1) Preparation of DNA-conjugated ethylene**

**Conditions of Step1:**

Head piece **HP** (1 equiv. 300 nmol, 300 μL, 1 mM in borate buffer (0.25 M in H_2_O, pH = 9.4)), then add the pre-mixture of **2-bromoarylic acid**(40 equiv., 1.2 μmol, 60 μL, 0.2 M in DMA), **NHS**(50 equiv., 1.5 μmol, 37.5 μL, 0.4 M in DMA), **DIC**(20 eq, 0.6 μmol, 15 μL, 0.4 M in DMA) are activated at 0 degree for 5 min. Half of the activation solution is added to DNA at 0 degree and reacted for 5 min. Next, the other half was added and reacted at 0 ^o^C degree for 5 min, and then reacted at 25 ^o^C for 20 min.

**Conditions of Step2:**

DNA conjugate **10** (1 equiv. 10 nmol, 10 μL, 1 mM in H_2_O), **Aryl boric acid** (100 equiv., 1 μmol, 5 μL, 0.2 M in DMSO), **sSPhos-Pd-G2** (2.5 equiv., 25 nmol, 2.5 μL, 10 mM in DMSO), **CsOH** (100 equiv., 1 μmol, 2 μL, 0.5 M in H_2_O), react at 80^o^C for 2 hrs. When time up, add **DDTC** (100 equiv., 1 μmol, 2.5 μL, 0.4 M in H_2_O), 80^o^C,10 min.

**Conditions of Step1:**

Head piece **HP** (1 equiv. 1 μmol, 1000 μL, 1 mM in borate buffer (0.25 M in H_2_O, pH = 9.4)), **acid** (50 equiv., 50 μmol, 250 μL, 0.2 M in DMA), **HATU** (50 equiv., 50 μmol, 125 μL, 0.4 M in DMA), **DIPEA** (50 equiv., 50 μmol, 125 μL, 0.4 M in DMA), premix the acid, HATU and DIPEA at 0^o^C for 5 min, then the mixture were took into DNA system, 25^o^C,1 hour.

**Conditions of Step2:**

DNA conjugates **7** (1 equiv. 10 nmol, 10 μL, 1 mM in H_2_O), **Pinacol vinylboronate** (10 equiv., 100 nmol, 0.5 μL, 0.2 M in DMSO), **sSPhos-Pd-G2** (2.5 equiv., 25 nmol, 2.5 μL, 10 mM in DMSO), **CsOH** (100 equiv., 1 μmol, 2 μL, 0.5 M in H_2_O), 100^o^C, 0.5 hour. When time up, and **DDTC** (100 equiv., 1 μmol, 2.5 μL, 0.4 M in H_2_O), 90^o^C,10 min.

**2) Condition optimization**

| Entry | **Deviation from std conditions**^[b]^ | **3a** | **4** | **1a** |
| --- | --- | --- | --- | --- |
| 1 | none | 88 | <5 | 6 |
| 2 | No Pd(OAc)_2_ | 6 | <5 | 85 |
| 3 | No Pd(OAc)_2_, 16 hours | 10 | <5 | 82 |
| 4 | PdCl_2_ | 72 | <5 | 20 |
| 5 | Pd(PPh_3_)_4_ | 61 | <5 | 27 |
| 6 | 200 equiv. **2a**, 0.4M in DMSO, 116mM | 65 | <5 | 28 |
| 7 | 100 equiv. **2a**, 0.2M in DMSO, 58mM | 49 | 6 | 42 |
| 8 | no CsOH, pH~9 | 55 | <5 | 39 |
| 9 | 500 equiv. CsOH, 5 M in H_2_O, 290mM, pH~12 | 48 | 32 | 11 |
| 10 | 100 nmol DNA | 86 | <5 | 8 |

Table S1. Condition optimization

^[a]^ Reaction conditions: DNA **1a** (10 nmol, 10μL 0.25M borate buffer (pH=9.4), 1 equiv.), **2a** (500 equiv., 1 M in DMSO, 5 μL), CsOH (100 equiv., 1 M in H2O, 1 μL); Pd catalyst (2.5 equiv., 20 mM in DMSO, 1.25 μL), pH~10.5, DMSO/H_2_O=25/44, (concentration of DNA conjugate **1a**: 0.58mM; **2a**: 290mM; CsOH: 58mM; Pd(OAc)_2_: 1.4mM), 80 ^o^C,2 hours. The conversion was determined by LC-MS. [b] All deviation condition should compensate with corresponding solvent to keep identical DMSO/H2O ratio of reaction system as standard conditions.

1. Scope study of amines

Reaction condition: DNA (10 nmol, 1 mM in 0.25 M borote buffer, 1 equiv); amine (1 M in DMSO, 500 equiv); CsOH (1 M in H2O, 100 equiv); Pd catalyst (40 mM in DMSO, 2.5 equiv); 80 ^o^C, 2 h. the conversions were determined by LC-MS

**The structures of target product were listed as follow**

**Table S2. Scope study of amine**

| Substrate | Structure | Expected MW | Observed MW | Conversion |
| --- | --- | --- | --- | --- |
| **3a** |  | 5374 | 5374.3 | 88% |
| **3b** |  | 5400 | 5399 | 92% |
| **3c** |  | 5471 | 5470.9 | 61% |
| **3d** |  | 5436 | 5435.5 | 94% |
| **3e** |  | 5422 | 5421.6 | 94% |
| **3f** |  | 5388 | 5388.1 | 95% |
| **3g** |  | 5497 | 5497.8 | 78% |
| **3h** |  | 5480 | 5479.7 | 52% |
| **3i** |  | 5498 | 5498.1 | 13% |
| **3j** |  | 5466 | 5465.5 | 22% |
| **3k** |  | 5386 | 5384.7 | 99% |
| **3l** |  | 5400 | 5399 | 99% |
| **3m** |  | 5415 | 5416.4 | 89% |
| **3n** |  | 5428 | 5428.1 | 52% |
| **3o** |  | 5469 | 5469.5 | 95% |
| **3p** |  | 5454 | 5454.4 | 95% |
| **3q** |  | 5469 | 5469.4 | 70% |
| **3r** |  | 5479 | 5479.3 | 95% |
| **3s** |  | 5448 | 5448.3 | 97% |
| **3t** |  | 5402 | 5403.2 | 38% |
| **3u** |  | 5428 | 5428.1 | 28% |
| **3v** |  | 5430 |  | <5% |
| **3w** |  | 5444 | 5443.1 | 87% |
| **3x** |  | 5458 | 5457.8 | 73% |
| **3y** |  | 5501 | 5501.4 | 77% |
| **3z** |  | 5503 | 5503.8 | 85% |
| **3aa** |  | 5468 | 5469.2 | 53% |
| **3ab** |  | 5515 | 5515.5 | 40% |
| **3ac** |  | 5408 |  | <5% |
| **3ad** |  | 5422 |  | <5% |
| **3ae** |  | 5438 | 5437.4 | <5% |
| **3af** |  | 5433 |  | <5% |

1. Scope study on DNA conjugated aryl ethylene

Reaction condition: DNA (10 nmol, 1 mM in 0.25 M borote buffer, 1 equiv); amine (1 M in DMSO, 500 equiv); CsOH (1 M in H2O, 100 equiv); Pd catalyst (40 mM in DMSO, 2.5 equiv); 80 ^o^C, 2 h. the conversions were determined by LC-MS

**The structures of target product were listed as follow**

**Table S3. Scope study on DNA conjugated aryl ethylene**

| Substrate | Structure | Expected MW | Observed MW | Conversion |
| --- | --- | --- | --- | --- |
| **3b** |  | 5400 | 5399 | 92% |
| **3ag** |  | 5401 | 5399.9 | 92% |
| **3ah** |  | 5390 | 5390 | 75% |
| **3ai** |  | 5401 | 5399.9 | 63% |
| **3aj** |  | 5440 | 5439.7 | 82% |
| **3ak** |  | 5468 | 5468.8 | 72% |
| **3al** |  | 5400 | 5399.7 | 45% |
| **3am** |  | 5400 | 5399.7 | 89% |
| **3an** |  | 5400 | 5399.4 | 58% |
| **3ao** |  | 5477 | 5477.3 | 40% |
| **3ap** |  | 5467 | 5467 | 74% |
| **3aq** |  | 5400 | 5399.4 | 60% |
| **3ar** |  | 5418 | 5418.2 | 50% |
| **3as** |  | 5399 | 5399 | 67% |
| **3at** |  | 5429 | 5429.7 | 51% |
| **3au** |  | 5364 | 5362.6 | 62% |
| **3av** |  | 5414 | 5413 | 67% |
| **3aw** |  | 5442 | 5442.3 | 63% |
| **3ax** |  | 5476 | 5475.5 | 95% |
| **3ay** |  | 5454 | 5453.5 | 64% |
| **3az** |  | 5512 | 5512.1 | 36% |
| **3ba** |  | 5472 | 5473.3 | 73% |
| **3bb** |  | 5522 | 5522.2 | 83% |
| **3bc** |  | 5455 | 5455 | 86% |
| **3bd** |  | 5392 | 5391.4 | 41% |
| **3be** |  | 5403 | 5402.6 | 48% |
| **3bf** |  | 5442 | 5442.2 | 38% |
| **3bg** |  | 5468 | 5468.7 | 39% |
| **3bh** |  | 5429 | 5428.3 | 47% |
| **3bi** |  | 5418 | 5417.3 | 41% |

1. DNA damage and qPCR evaluation

**Figure S4**. Reaction scheme on long-chained DNA, reaction performed with standard conditions

**1) Reaction on long-chain DNA**


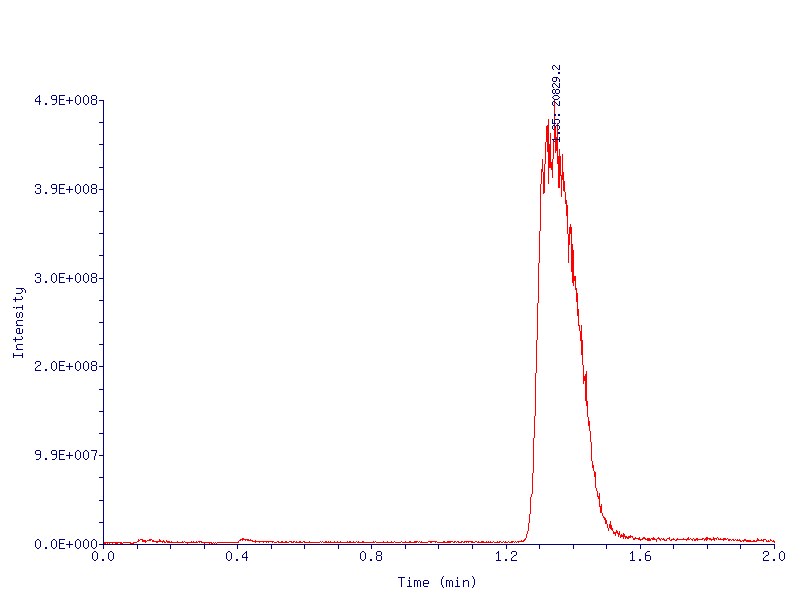


**Figure S5**. LC-MS spectrum of **C1-P1-HP-BB1**


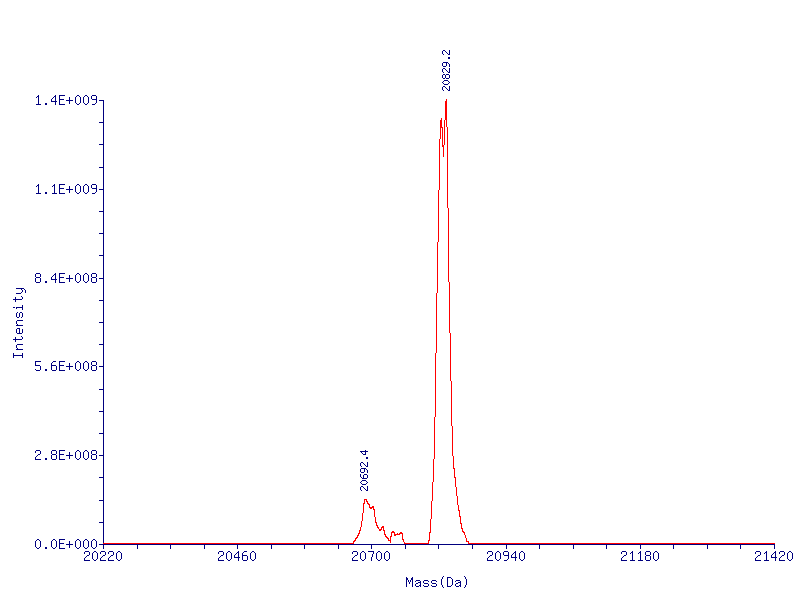


**Figure S6.** Deconvoluted mass spectrum of **C1-P1-HP-BB1**, expected: 20832, observed: 20829.2


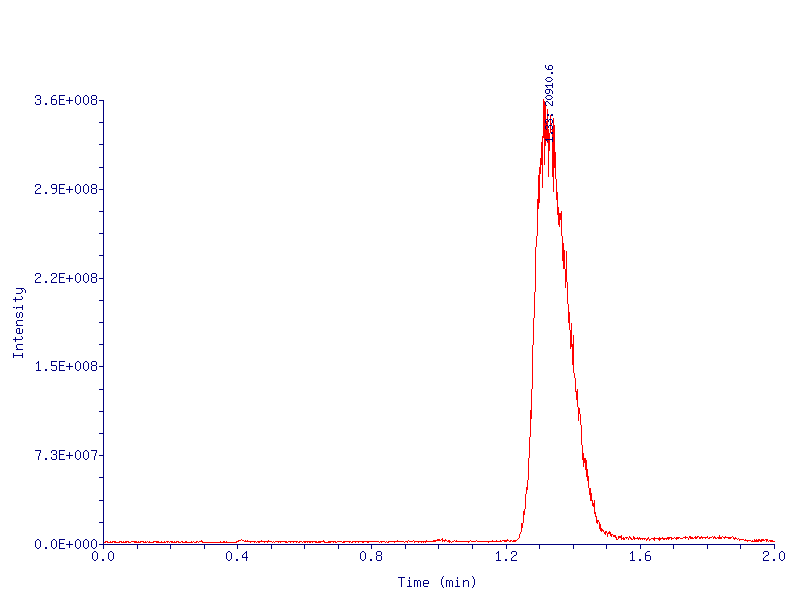


**Figure S7.** LC-MS spectrum of **C1-P1-HP-BB1-BB2**


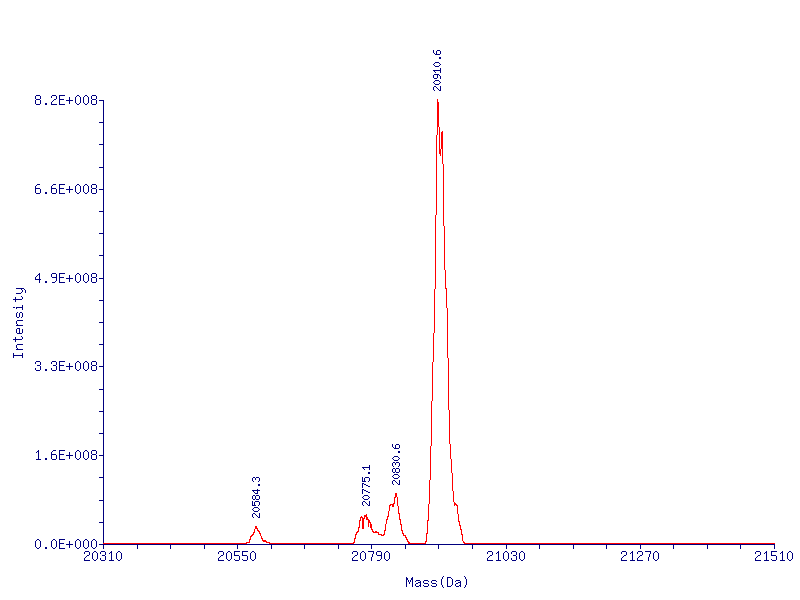


**Figure S8.** Deconvoluted mass spectrum of **C1-P1-HP-BB1-BB2**, expected: 20917, observed: 20910.6

**2) DNA Damage evaluation**

The DNA damage was evaluated by applying q-PCR test. Ligations without C-N formation reaction were used as a control experiment.

Ligation experiments followed the general procedures described above. Besides, further DNA magnetic beads purification was performed prior to qPCR test.


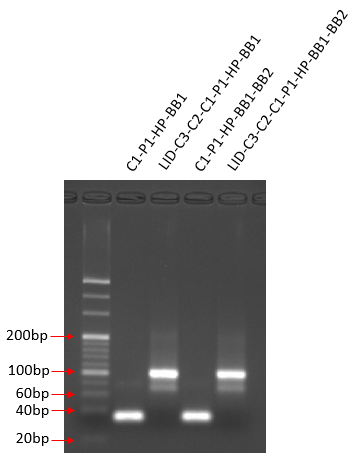


| Entry | Sample name | Gel yield |
| --- | --- | --- |
| 1 | LID-C3-C2-C1-P1-HP-BB1 | 82% |
| 2 | LID-C3-C2-C1-P1-HP-BB1-BB2 | 80% |

**Figure S9**. Reaction scheme for C2-C3-LID and gel results

**Total DNA quantification by Qubit.** The purified final products were quantified using Qubit dsDNA HS Assay Kit (Q32854, Thermo Fisher, MA, USA) and the Qubit 3.0 fluorometer (Thermo Fisher, MA, USA). Following the manufacturer’s instructions, samples are pre-diluted to a range of 0.2-20 ng/μl concentration (estimated by DNA input amount) by Tris buffer (10 mM, pH 8.0) for test. Concentrations provided by Qubit were used to calculate the molarity of the initial sample corrected by the dilution factor, and the purity of target DNA fragment.

**q-PCR test (Amplifiable DNA quantification).** The beads purified final products were quantified by qPCR (quantitative polymerase chain reaction) using a SYBR Green Master Mix kit (1804053, Life technologies, USA) and a StepOnePlus™ Real-Time PCR System (Thermo Sci., USA). Following the manufacturer’s guidelines, dilution series were made for all samples and run in parallel with an in-house made dsDNA standards (a series of pre-made dilutions of a 138 bp dsDNA fragment). All samples and standards were run in duplicates and subjected to PCR cycles as follows: 95 °C heat activation for 10 min followed by 30 cycles of 95 °C denaturation for 10 seconds, 55 °C annealing for 10 seconds and extension at 72 °C for 10 seconds. The standard curve was used to calculate the PCR efficiency and subsequently the molarity of the libraries after correction.

**Table S4:** qPCR measurement and DNA damage evaluation

| **Sample** | **Quantification by Qubit** (mM) | **Quantification by qPCR** (mM) | **Amplifiable DNA ratio** |
| --- | --- | --- | --- |
| Sample 1 | 0.0196 | 0.0180 | 91% |
| Sample 2 | 0.0214 | 0.0180 | 84% |

The results shown in Table S4 indicate the good stability and readability of each DNA tag treated under standard conditions.

1. Structural confirmation

**Synthesis of DNA conjugate 5.** 200 nmol HP-AOP-NH_2_ was dissolved in pH 9.4 borate buffer (250 mM) to make 1 mM solution. Stocks of 40 equiv. **4** (200 mM in DMA), 40 equiv. HATU (200 mM in DMA), and 40 equiv. DIPEA (200 mM in DMA) were cooled at 4 degree for 5 min, and then mixed together. After vortex, the "premix" stock was cooled at 4 degree for 5 min, then transferred to DNA solution, vortex again. The reaction was allowed to proceed at room temperature for 1 h.

**Synthesis of 3bj**: DNA **1b** (10 nmol, 1 mM in 0.25 M borote buffer, 1 equiv.); amine **B** (1 M in DMSO, 500 equiv.); CsOH (1 M in H2O, 100 equiv.); Pd catalyst (20 mM in DMSO, 2.5 equiv.); 80 ^o^C, 2 h.


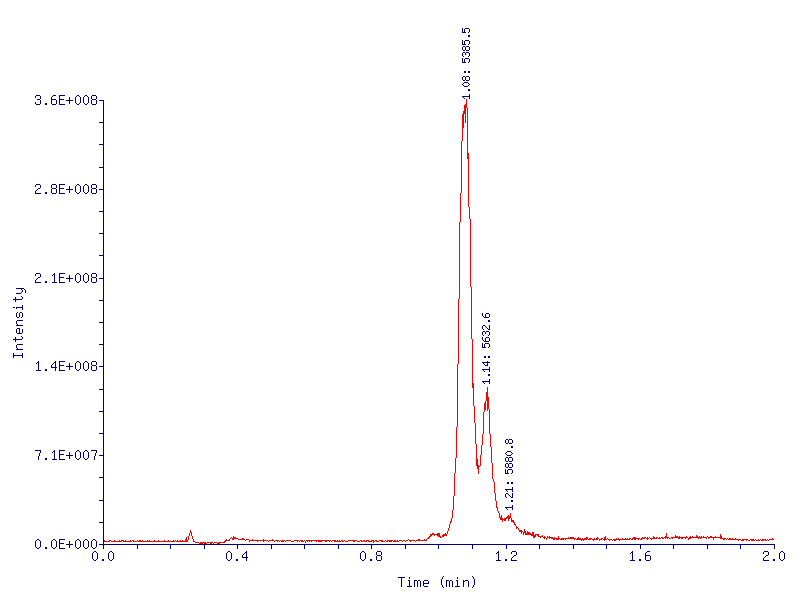


**Figure S10**. LC-MS spectrum of **5,** expected Mass: 5386; observed Mass: 5385.5


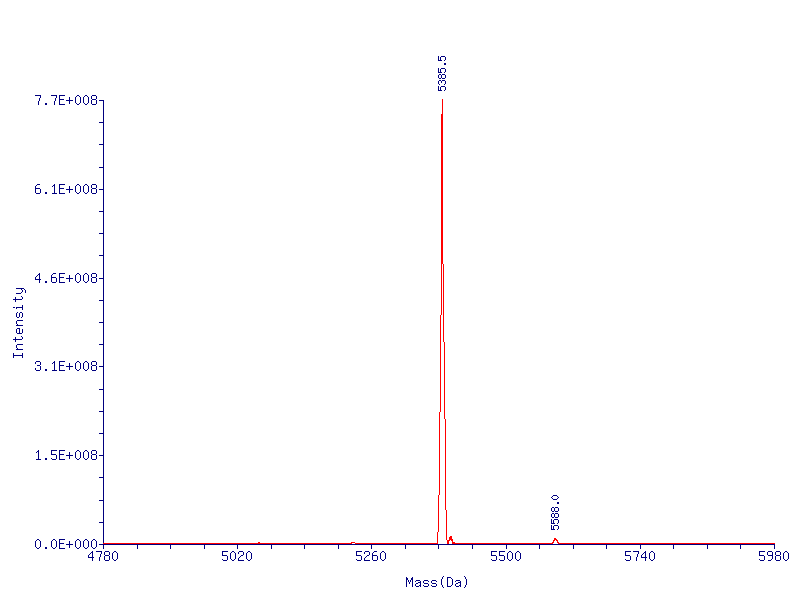


**Figure S11.** Deconvoluted mass spectrum of DNA conjugate **5**, expected: 5386; observed Mass: 5385.5


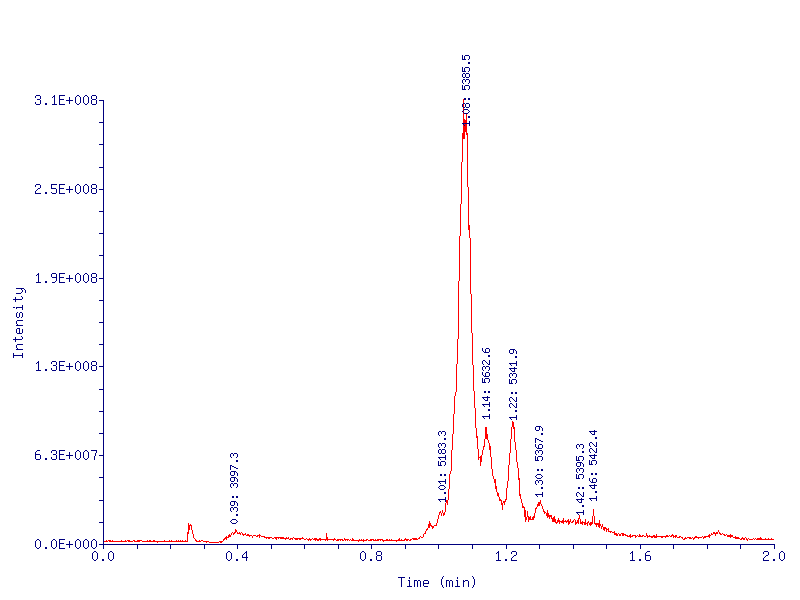


**Figure S12**. LC-MS spectrum of **3bj,** expected Mass: 5386; observed Mass: 5385.5


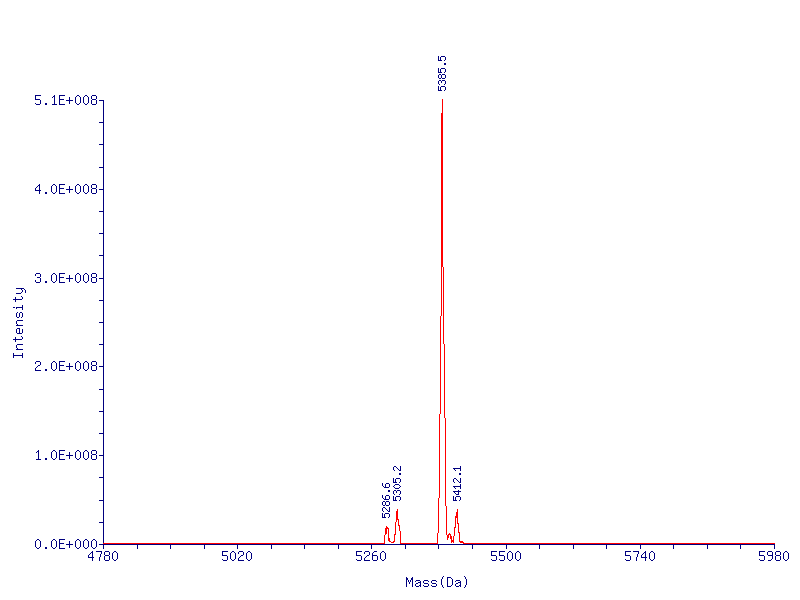


**Figure S13.** Deconvoluted mass spectrum of DNA conjugate **3bj**, expected: 5386; observed Mass: 5385.5


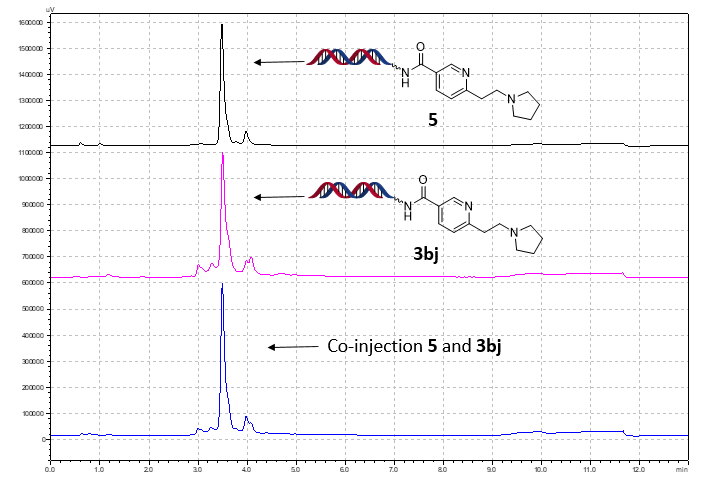


**Figure S14.** HPLC analysis of co-injection between **5** and **3bj**

**Figure S15.** ^1^H NMR of **4** in CD_3_OD

**Figure S16.** ^13^C NMR of **4** in D_2_O

**Off-DNA synthesis**

To an oven dried screw-cap reaction tube equipped with stir bar, were added **M1** (0.2 mmol), **2b** (10 mmol, 50 equiv.), Pd(OAc)_2_ (20 mol%), CsOH (0.5 mmol), followed by 2 mL DMSO and 1 mL H_2_O. The reaction mixture was heated at 80 ^o^C for 2 hours. When time is up, 10mL H_2_O was added, extracted with DCM (3 x 5 mL), organic layer was dried with Na_2_SO_4_, concentrated under vacuum and the product was purified by silica-gel column chromatography using PE : EA= 20 : 1 to 1 : 1 as eluent to obtain **M2** as yellow oil (38 mg, 77% yield). ^1^H NMR (600 MHz, Chloroform-*d*) δ 8.42 (d, 1H), 8.06 (s, 1H), 8.02 (brd, 1H), 7.28 (d, 1H), 3.12 (q, 1H), 3.03 (d, 3H), 2.93 (m, 4H), 2.06 – 1.94 (m, 1H), 1.88 – 1.83 (m, 2H), 1.71 – 1.65 (m, 2H), 1.53 (m, 2H), 1.34 (m, 2H). ^13^C NMR (151 MHz, Chloroform-*d*) δ 164.09, 149.76, 149.00, 147.11, 125.52, 121.26, 58.73, 47.49, 34.74, 31.83, 25.09, 22.98. HRMS(ESI): [M+H]^+^ m/z calcd. For C_14_H_22_N_3_O^+^ = 248.1757, found = 248.1761.

The results of off-DNA synthesis indicated that the on-DNA hydroamination protocol we established lead to product with desired structure.

**
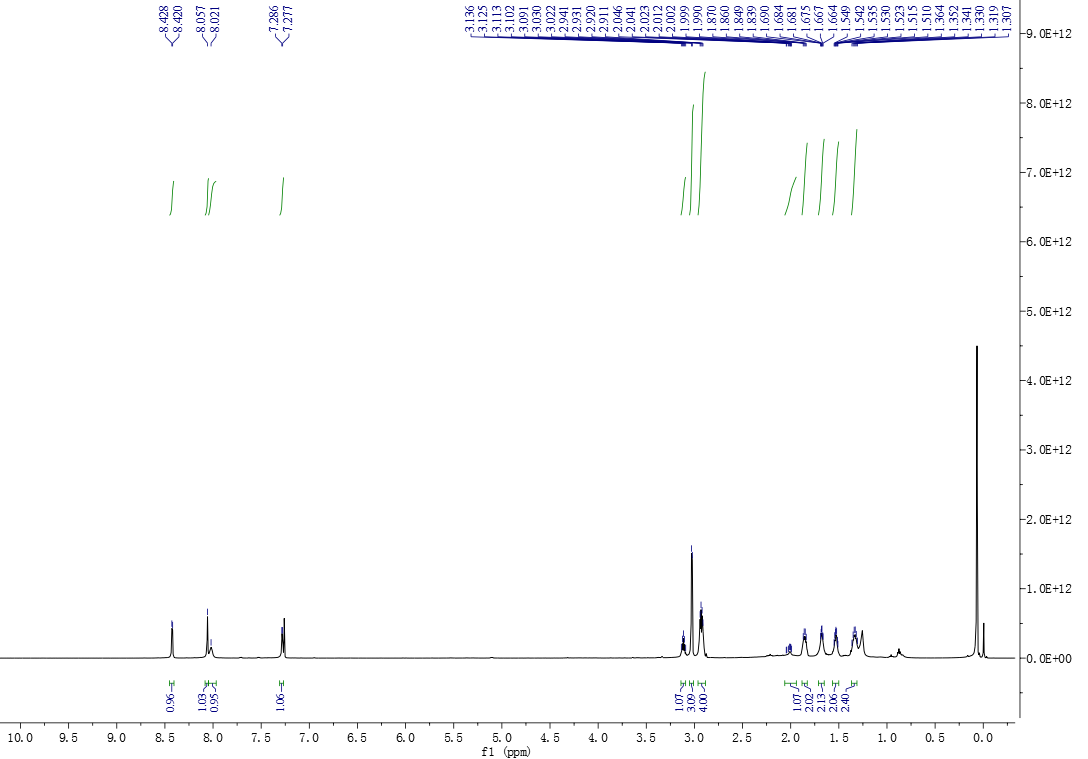
**

**Figure S17.** ^1^H NMR of **M2** in CDCl_3_

**
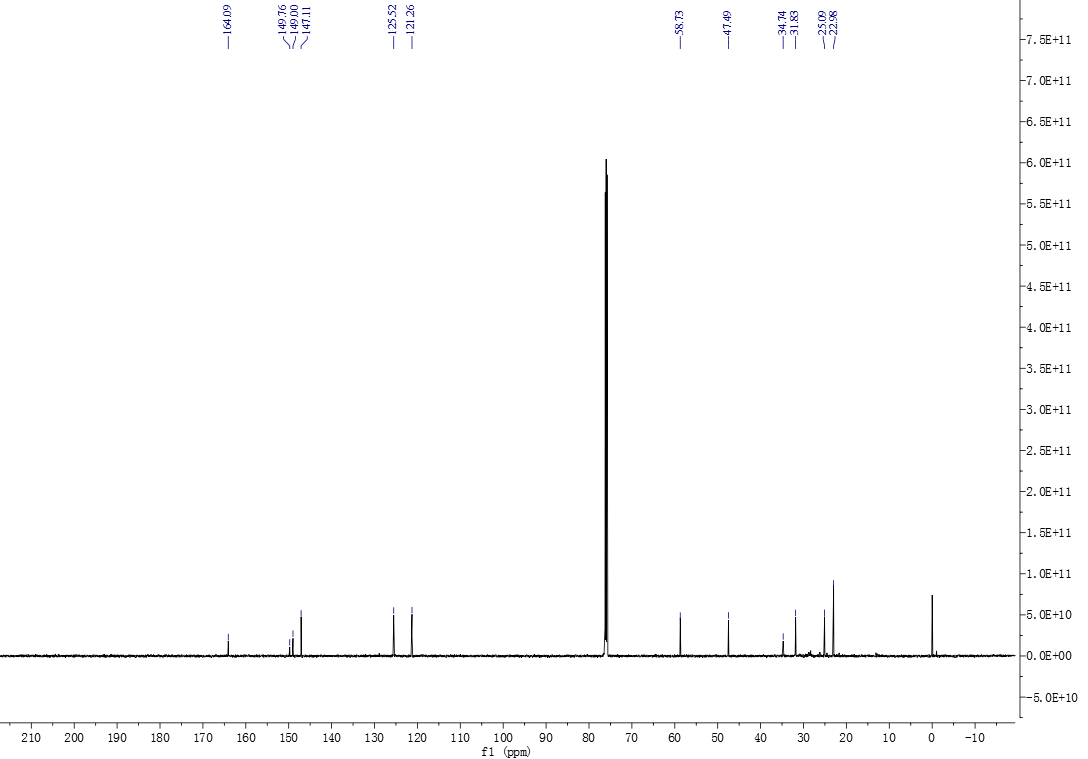
**

**Figure S18.** ^13^C NMR of **M2** in CDCl_3_

1. Deconvoluted Mass Spectra


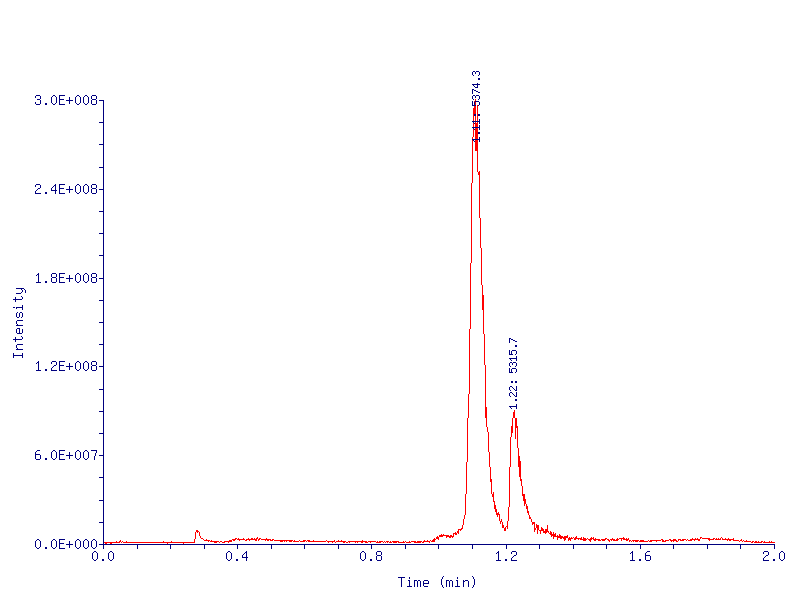


**Figure S19**. LC-MS spectrum of **3a,** expected Mass: 5374; observed Mass: 5374.3


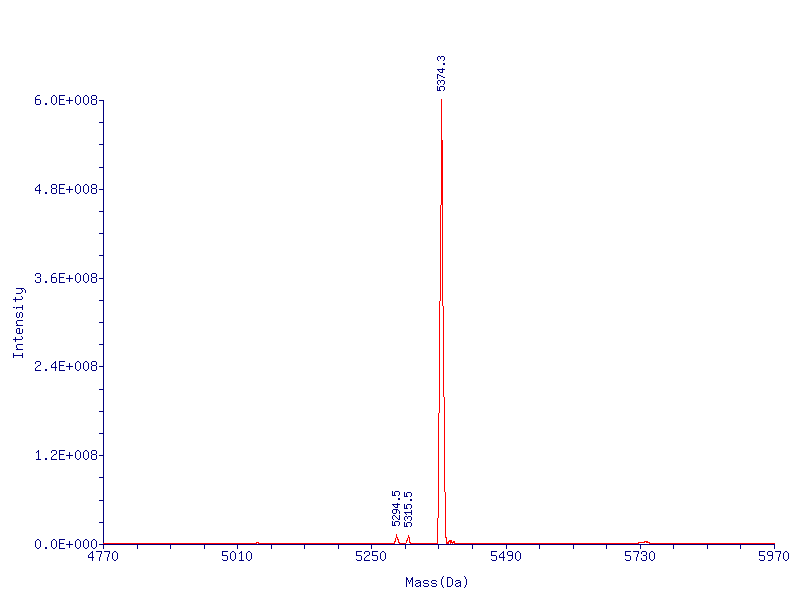


**Figure S20**. Deconvoluted mass spectrum of **3a,** expected Mass: 5374; observed Mass: 5374.3


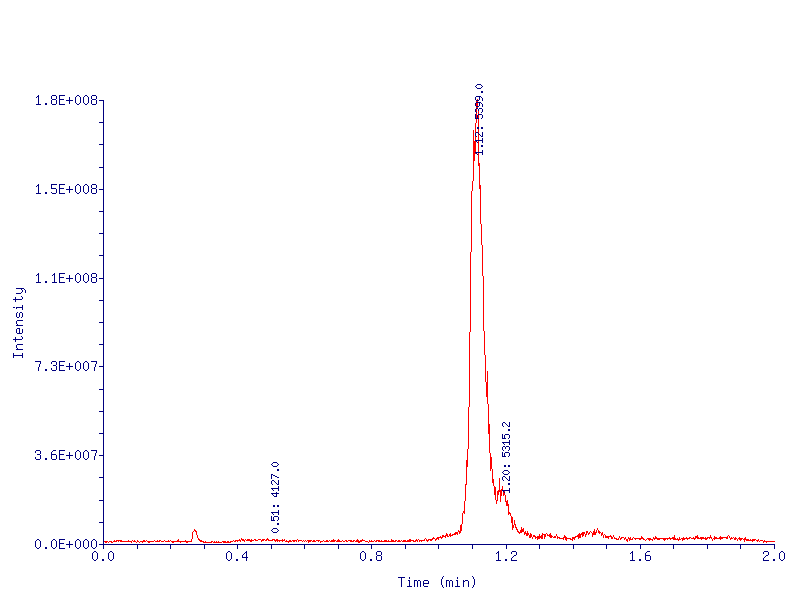


**Figure S21**. LC-MS spectrum of **3b,** expected Mass: 5400; observed Mass: 5399


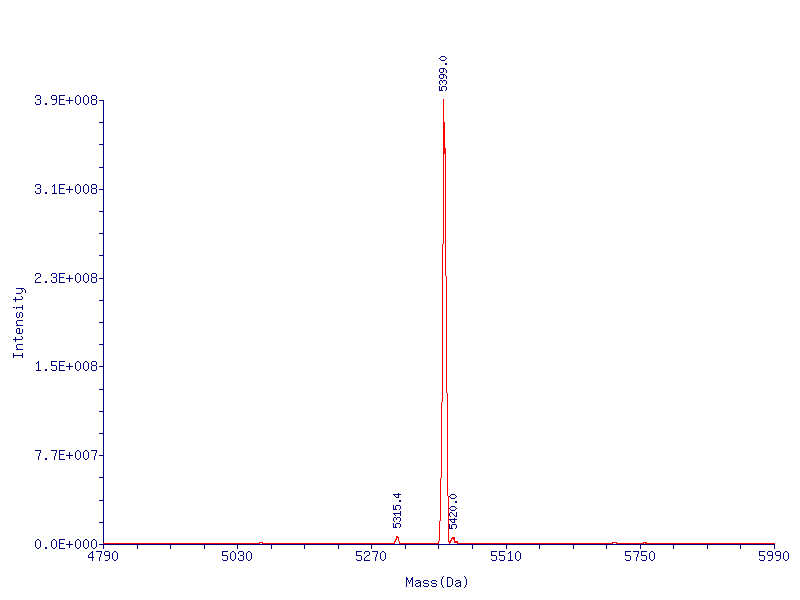


**Figure S22**. Deconvoluted mass spectrum of **3b,** expected Mass: 5400; observed Mass: 5399


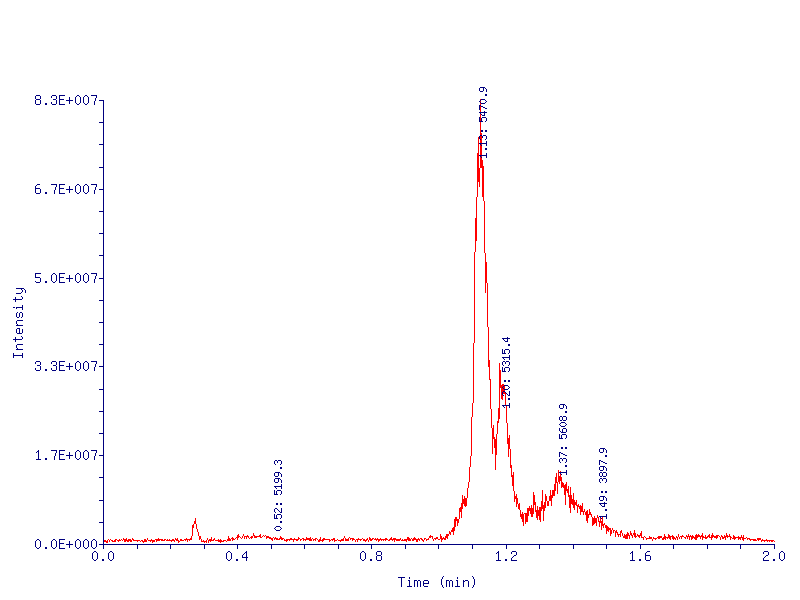


**Figure S23**. LC-MS spectrum of **3c,** expected Mass: 5471; observed Mass: 5470.9


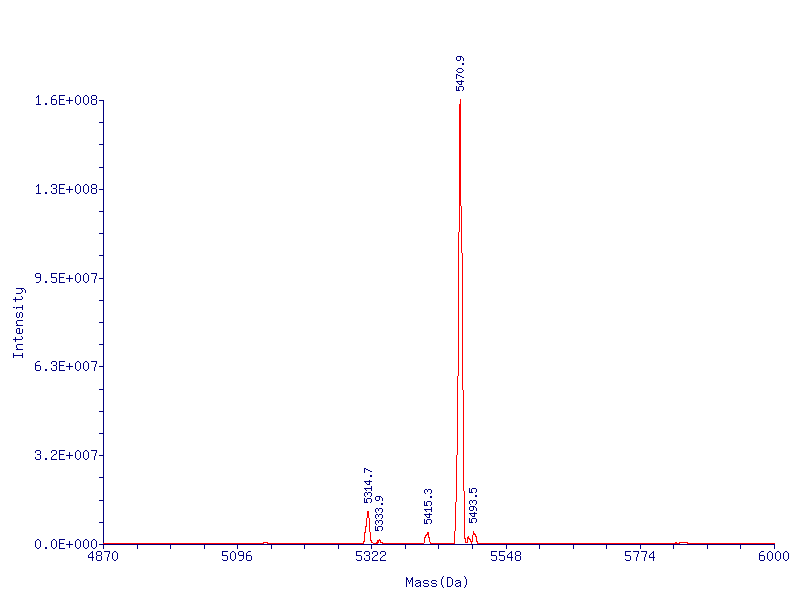


**Figure S24**. Deconvoluted mass spectrum of **3c,** expected Mass: 5471; observed Mass: 5470.9


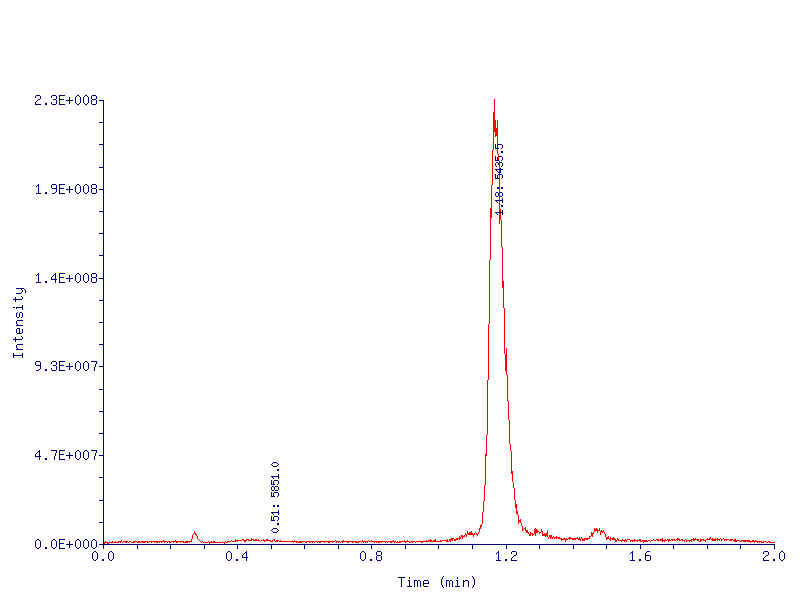


**Figure S25**. LC-MS spectrum of **3d,** expected Mass: 5436; observed Mass: 5435.5


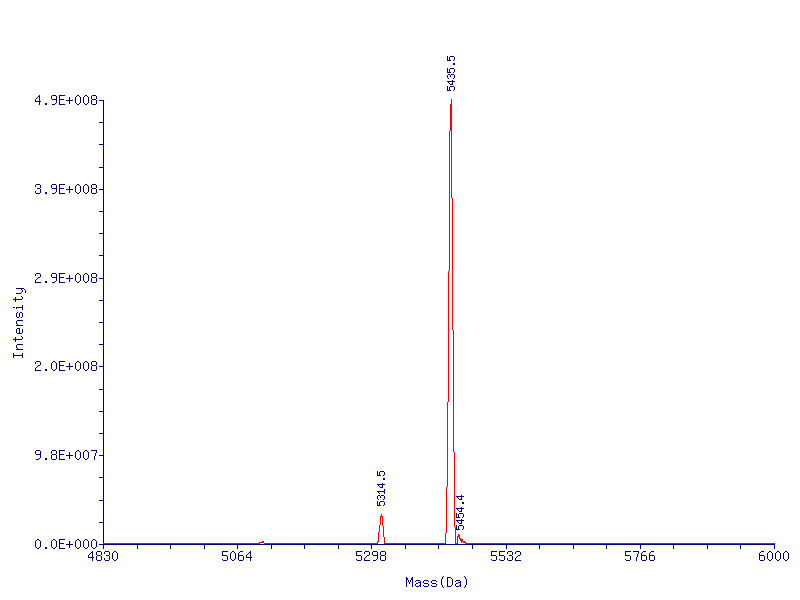


**Figure S26**. Deconvoluted mass spectrum of **3d,** expected Mass: 5436; observed Mass: 5435.5


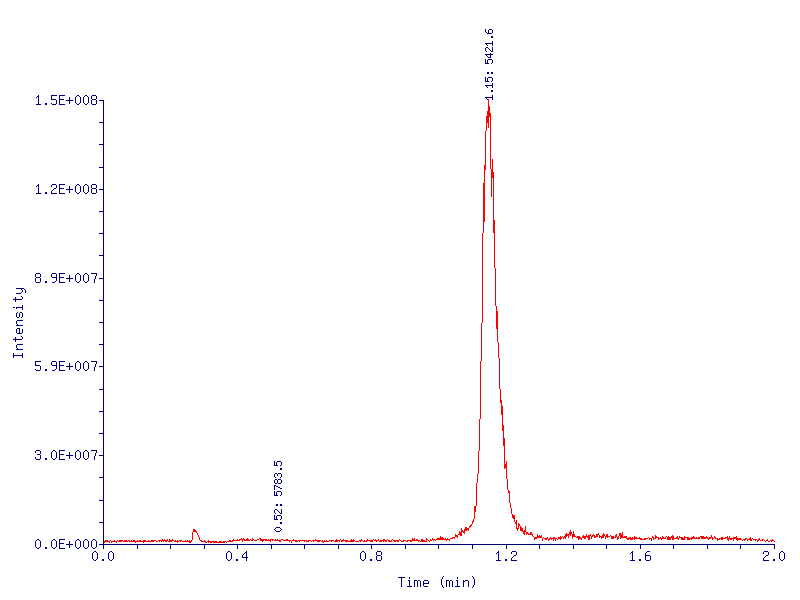


**Figure S27**. LC-MS spectrum of **3e,** expected Mass: 5422; observed Mass: 5421.6


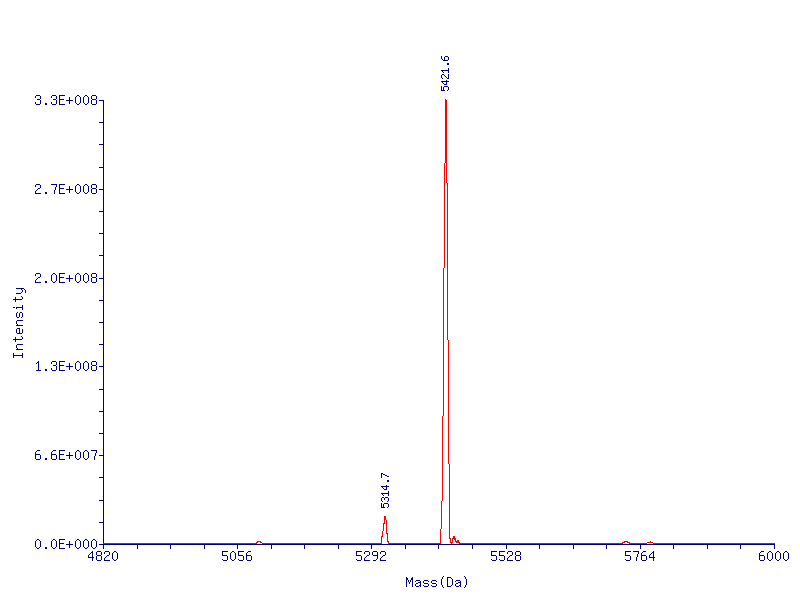


**Figure S28**. Deconvoluted mass spectrum of **3e,** expected Mass: 5422; observed Mass: 5421.6


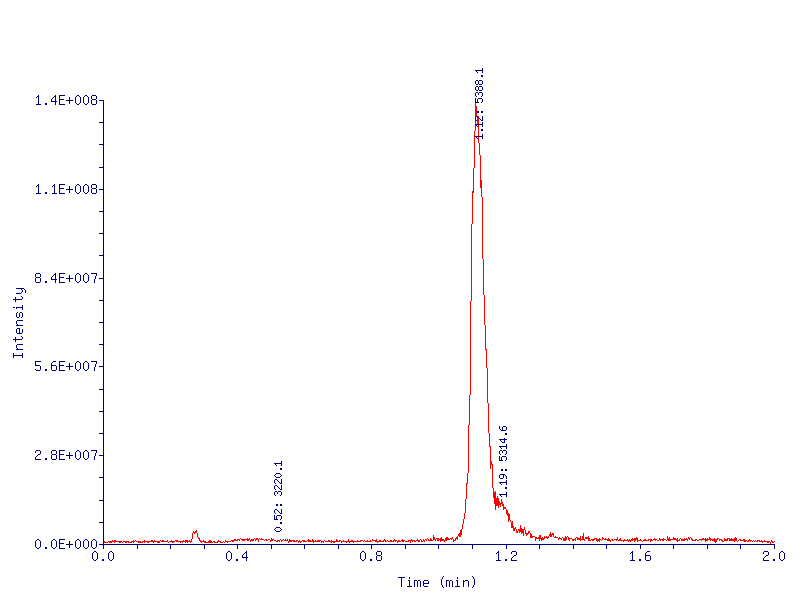


**Figure S29**. LC-MS spectrum of **3f,** expected Mass: 5388; observed Mass: 5388.1


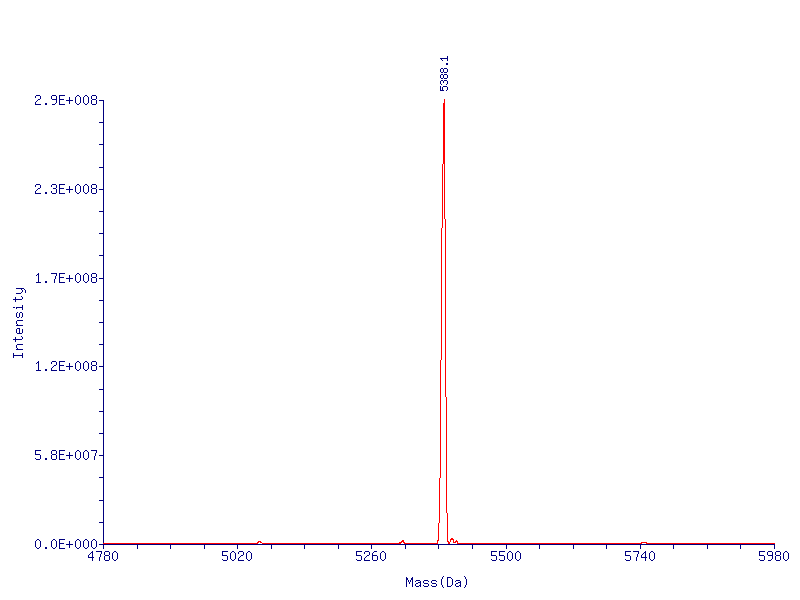


**Figure S30**. Deconvoluted mass spectrum of **3f,** expected Mass: 5388; observed Mass: 5388.1


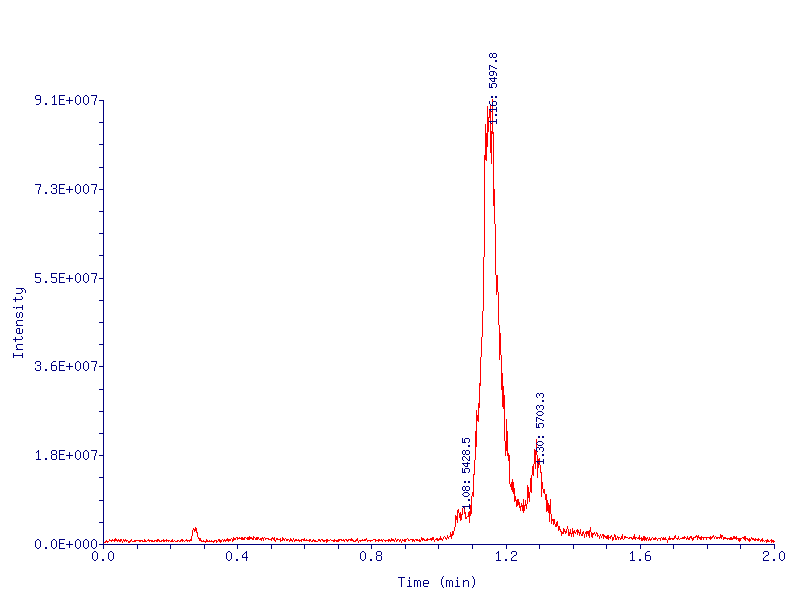


**Figure S31**. LC-MS spectrum of **3g,** expected Mass: 5497; observed Mass: 5497.8


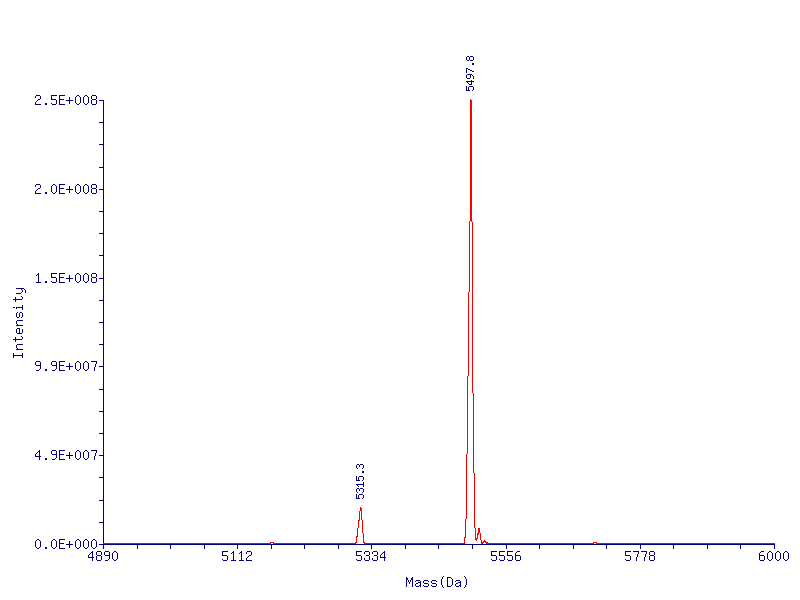


**Figure S32**. Deconvoluted mass spectrum of **3g,** expected Mass: 5497; observed Mass: 5497.8


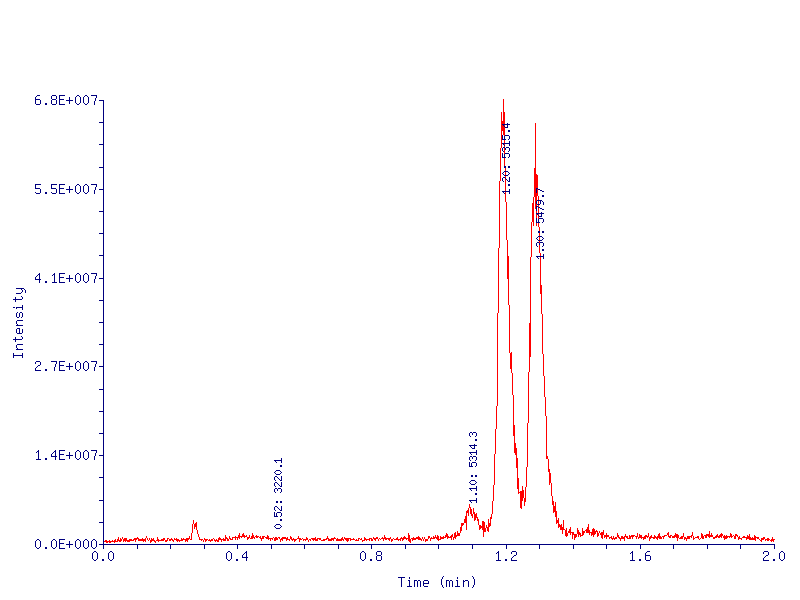


**Figure S33**. LC-MS spectrum of **3h,** expected Mass: 5480; observed Mass: 5479.7


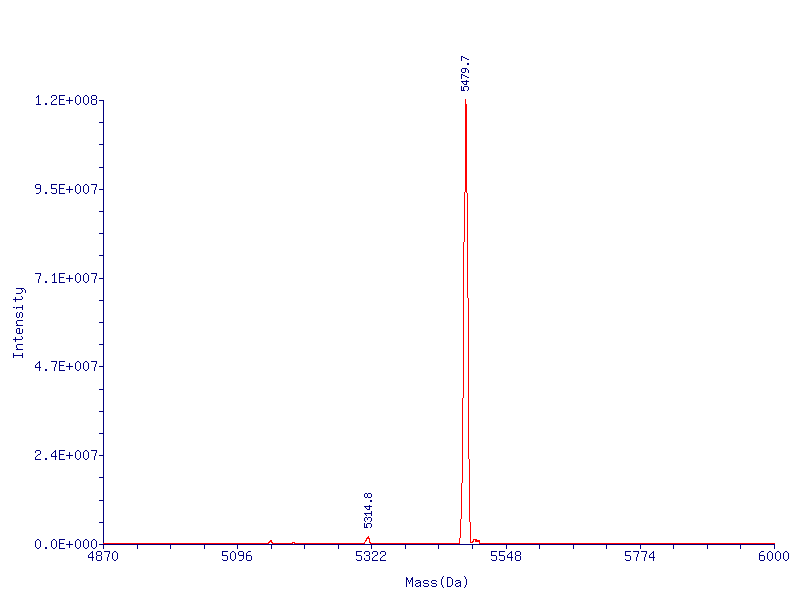


**Figure S34**. Deconvoluted mass spectrum of **3h,** expected Mass: 5480; observed Mass: 5479.7


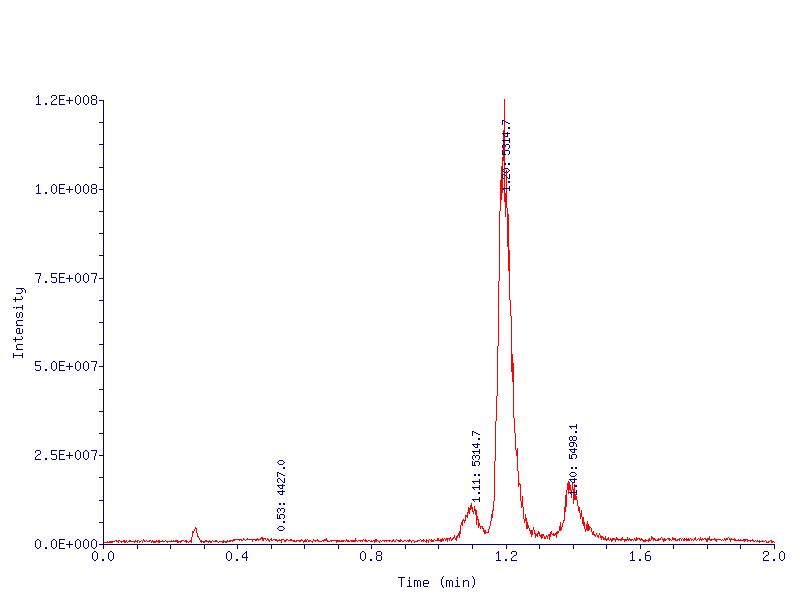


**Figure S35**. LC-MS spectrum of **3i,** expected Mass: 5498; observed Mass: 5498.1


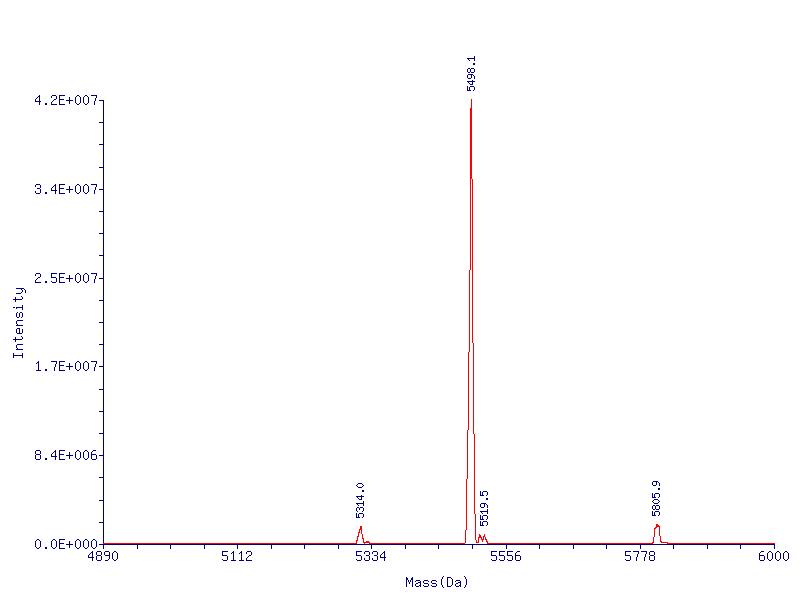


**Figure S36**. Deconvoluted mass spectrum of **3i,** expected Mass: 5498; observed Mass: 5498.1


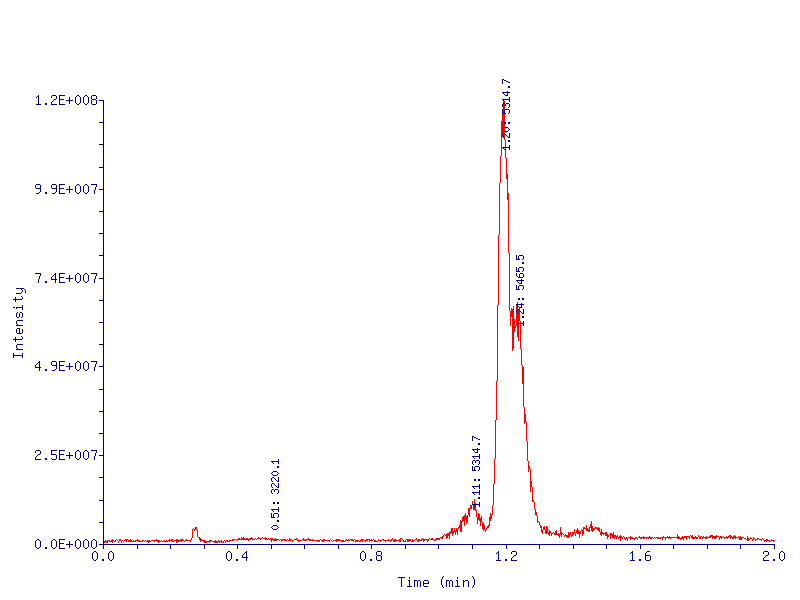


**Figure S37**. LC-MS spectrum of **3j,** expected Mass: 5466; observed Mass: 5465.5


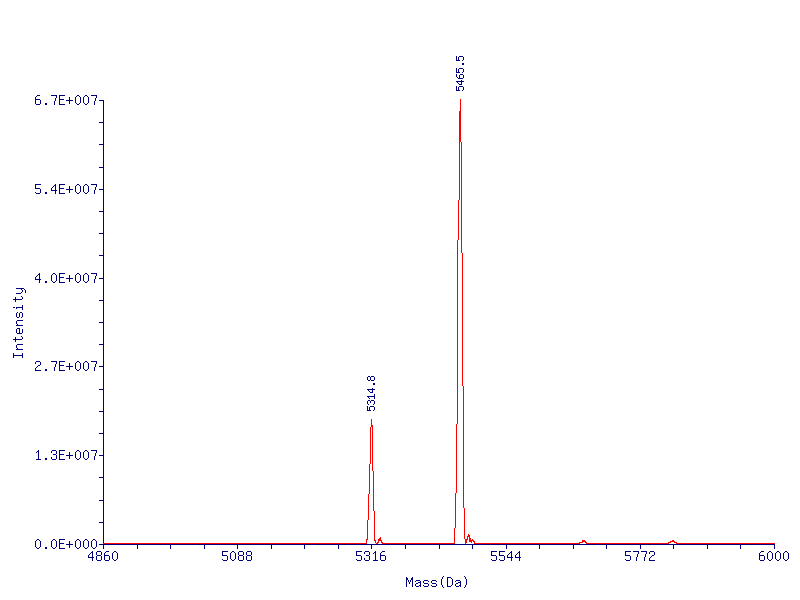


**Figure S38**. Deconvoluted mass spectrum of **3j,** expected Mass: 5466; observed Mass: 5465.5


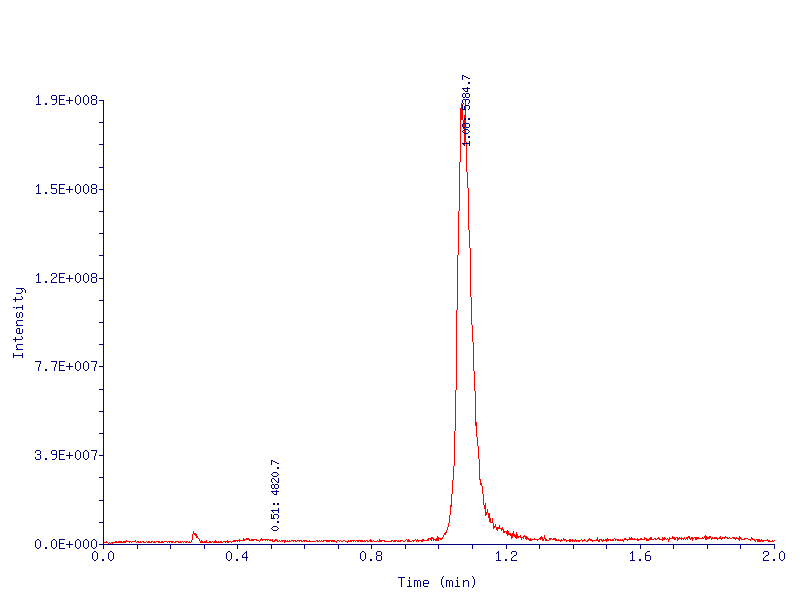


**Figure S39**. LC-MS spectrum of **3k,** expected Mass: 5386; observed Mass: 5384.7


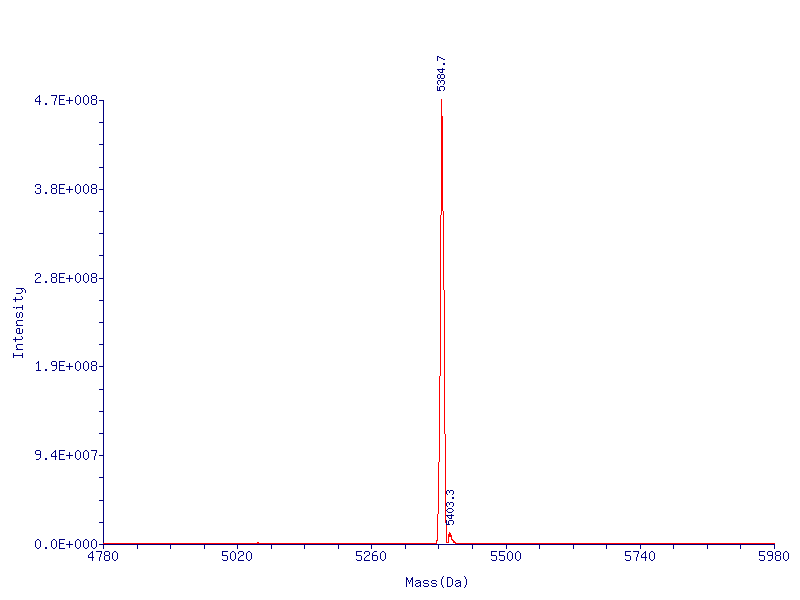


**Figure S40**. Deconvoluted mass spectrum of **3k,** expected Mass: 5386; observed Mass: 5384.7


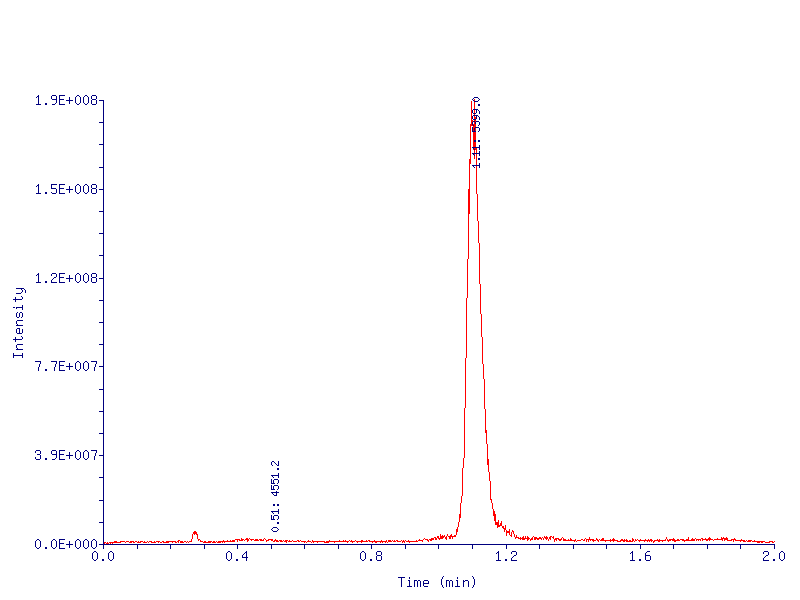


**Figure S41**. LC-MS spectrum of **3l,** expected Mass: 5400; observed Mass: 5399


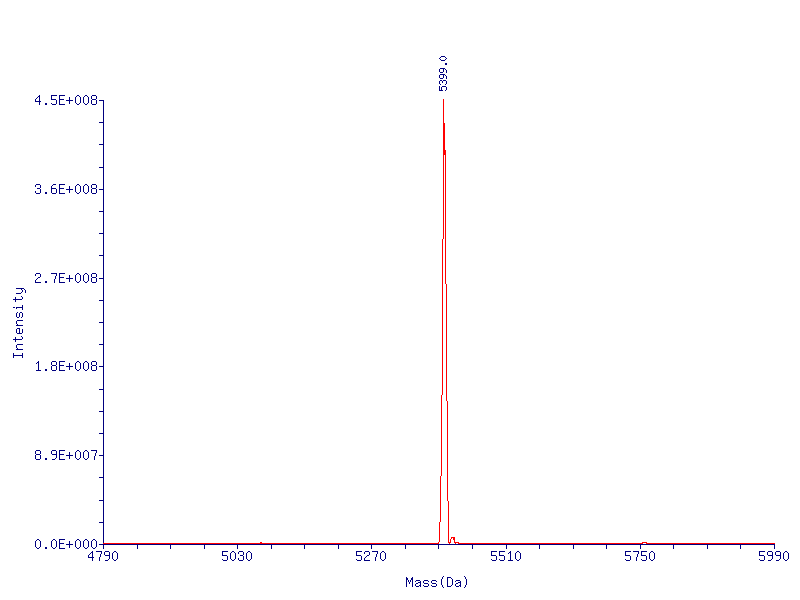


**Figure S42**. Deconvoluted mass spectrum of **3l,** expected Mass: 5400; observed Mass: 5399


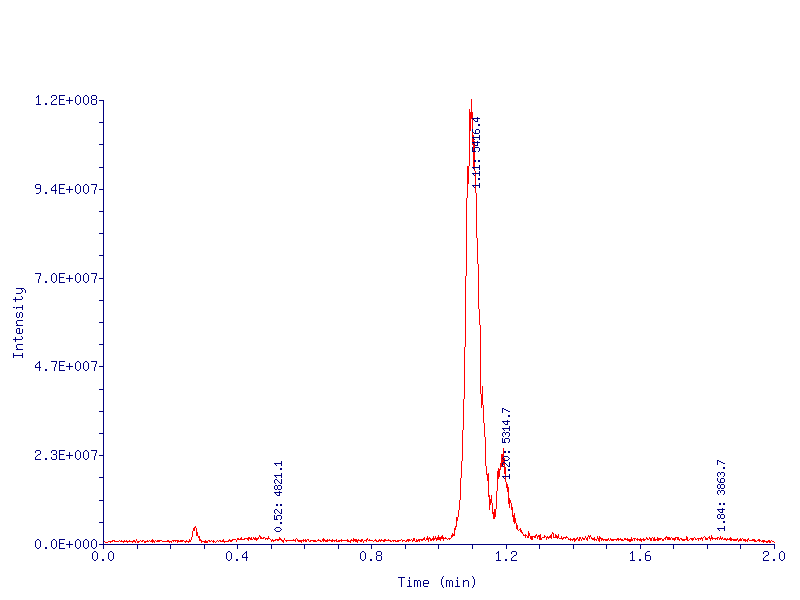


**Figure S43**. LC-MS spectrum of **3m,** expected Mass: 5415; observed Mass: 5416.4


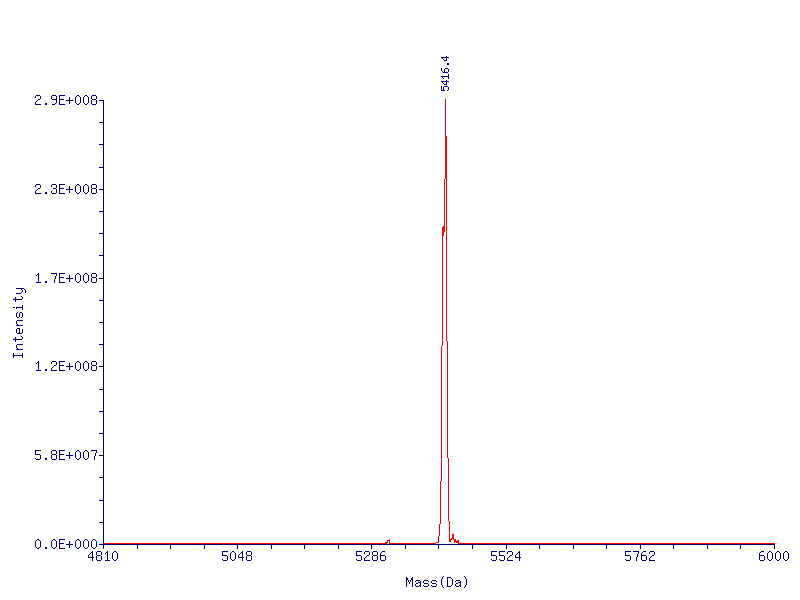


**Figure S44**. Deconvoluted mass spectrum of **3m,** expected Mass: 5415; observed Mass: 5416.4


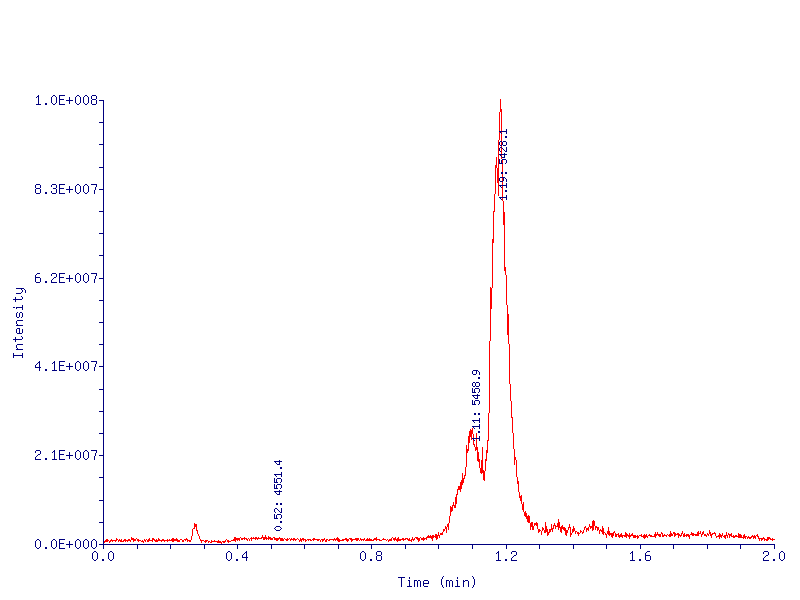


**Figure S45**. LC-MS spectrum of **3n,** expected Mass: 5428; observed Mass: 5428.1


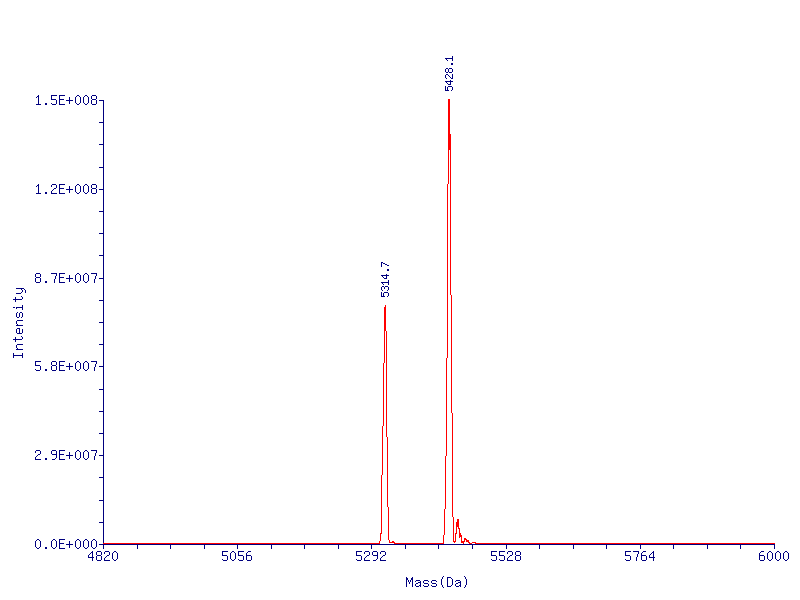


**Figure S46**. Deconvoluted mass spectrum of **3n,** expected Mass: 5428; observed Mass: 5428.1


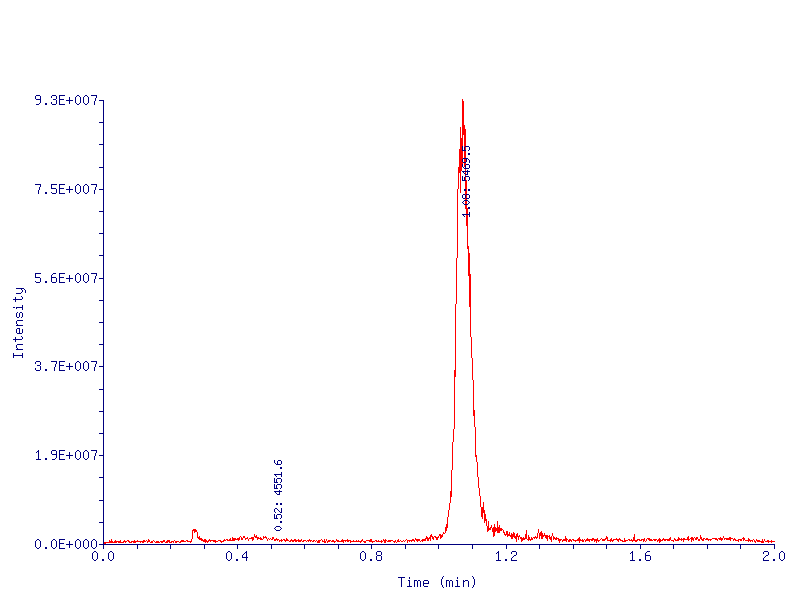


**Figure S47**. LC-MS spectrum of **3o,** expected Mass: 5469; observed Mass: 5469.5


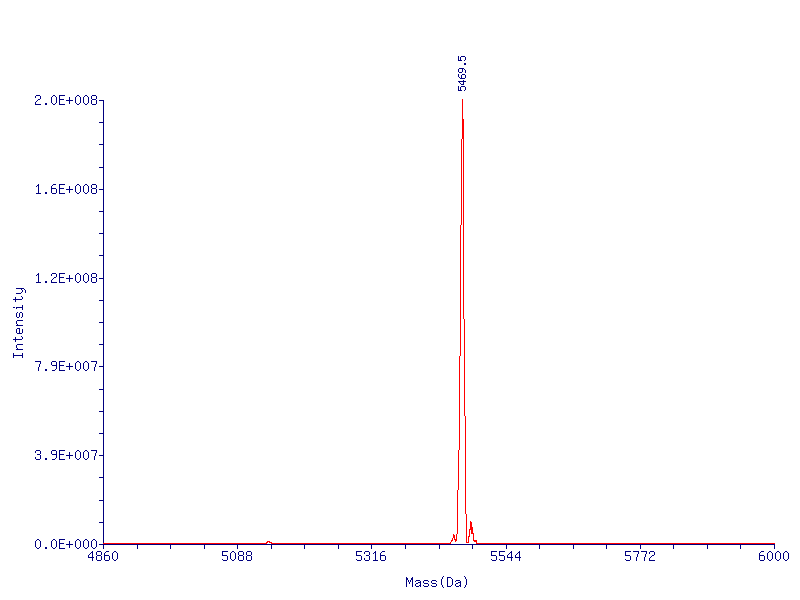


**Figure S48**. Deconvoluted mass spectrum of **3o,** expected Mass: 5469; observed Mass: 5469.5


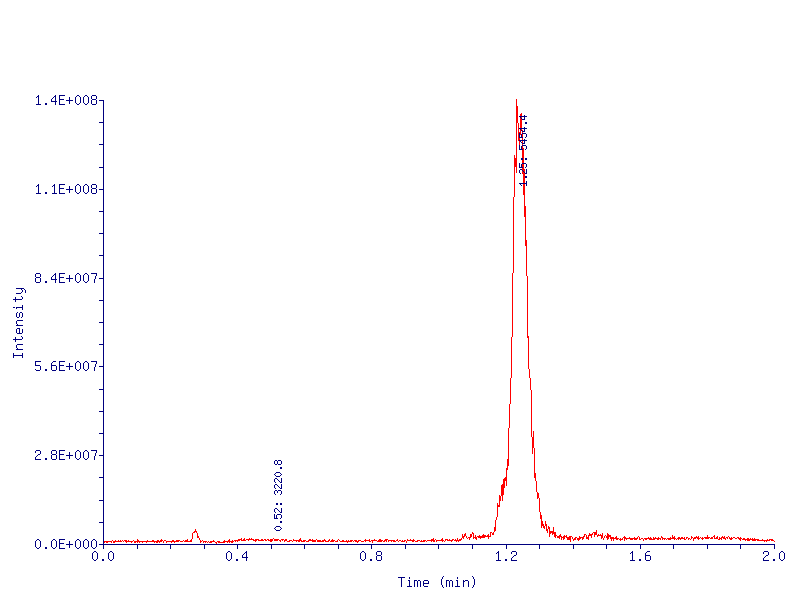


**Figure S49**. LC-MS spectrum of **3p,** expected Mass: 5454; observed Mass: 5454.4


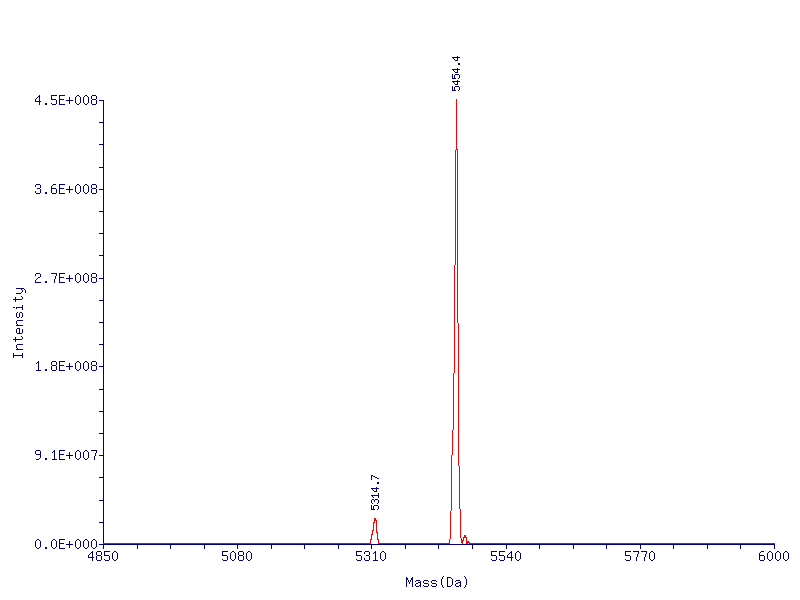


**Figure S50**. Deconvoluted mass spectrum of **3p,** expected Mass: 5454; observed Mass: 5454.4


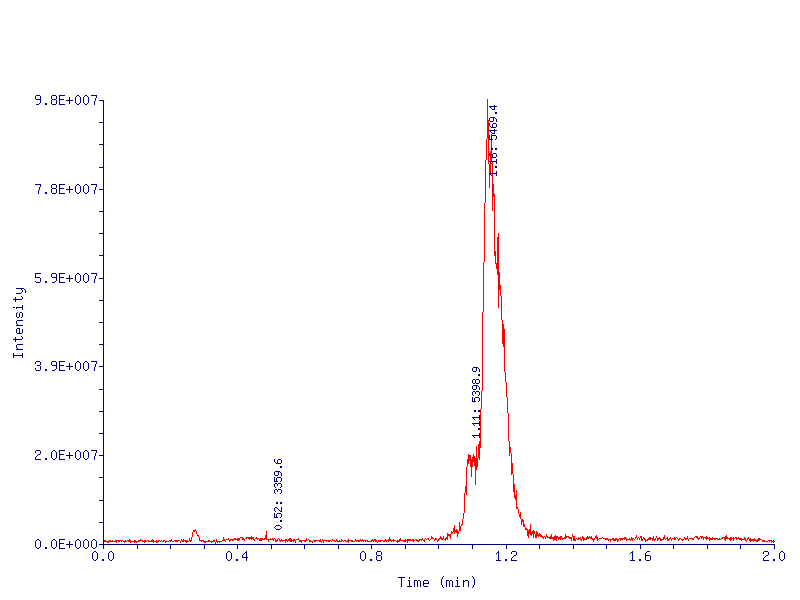


**Figure S51**. LC-MS spectrum of **3q,** expected Mass: 5469; observed Mass: 5469.4


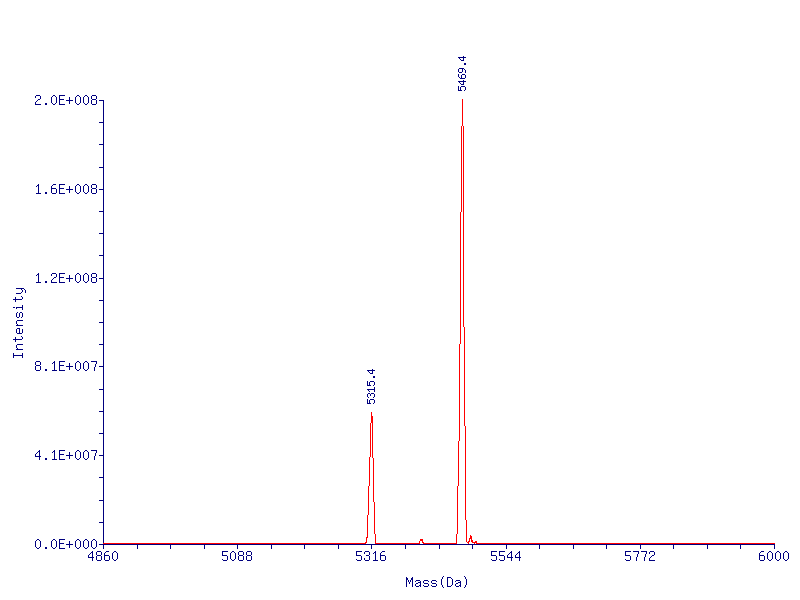


**Figure S52**. Deconvoluted mass spectrum of **3q,** expected Mass: 5469; observed Mass: 5469.4


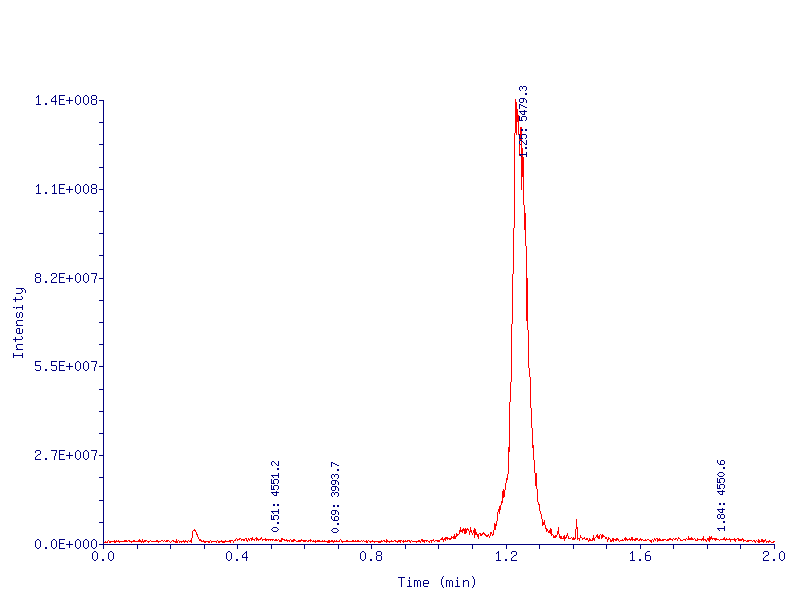


**Figure S53**. LC-MS spectrum of **3r,** expected Mass: 5479; observed Mass: 5479.3


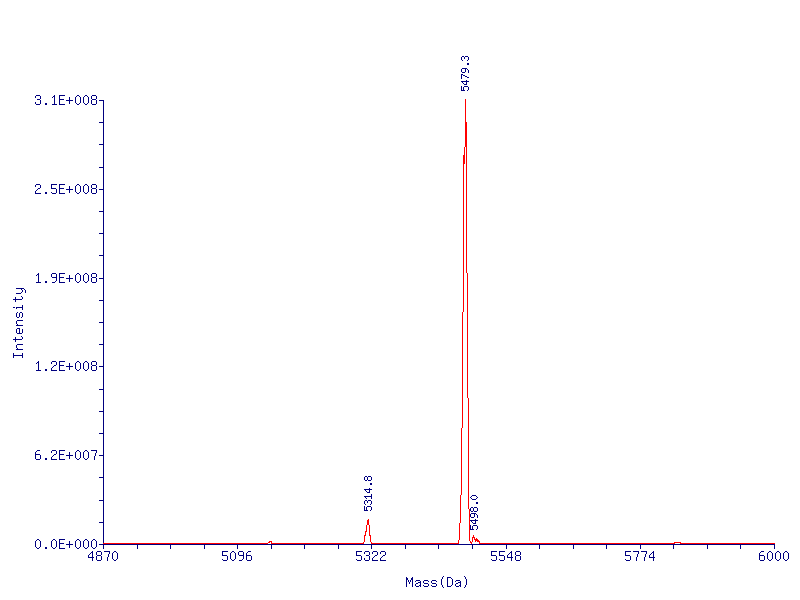


**Figure S54**. Deconvoluted mass spectrum of **3r,** expected Mass: 5479; observed Mass: 5479.3


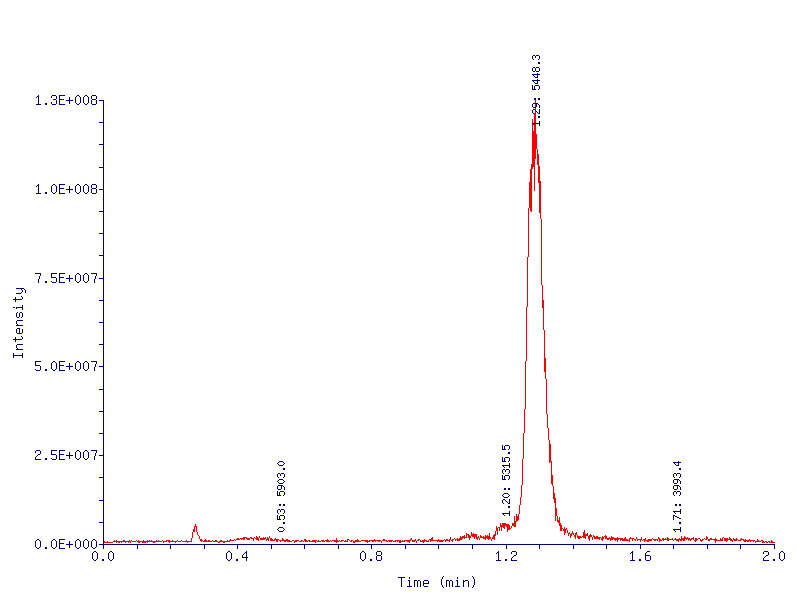


**Figure S55**. LC-MS spectrum of **3s,** expected Mass: 5448; observed Mass: 5448.3


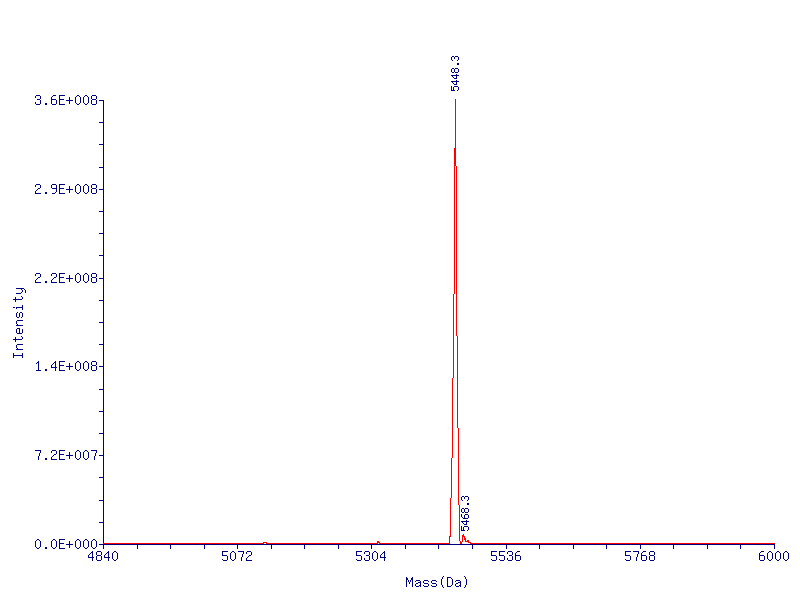


**Figure S56**. Deconvoluted mass spectrum of **3s,** expected Mass: 5448; observed Mass: 5448.3


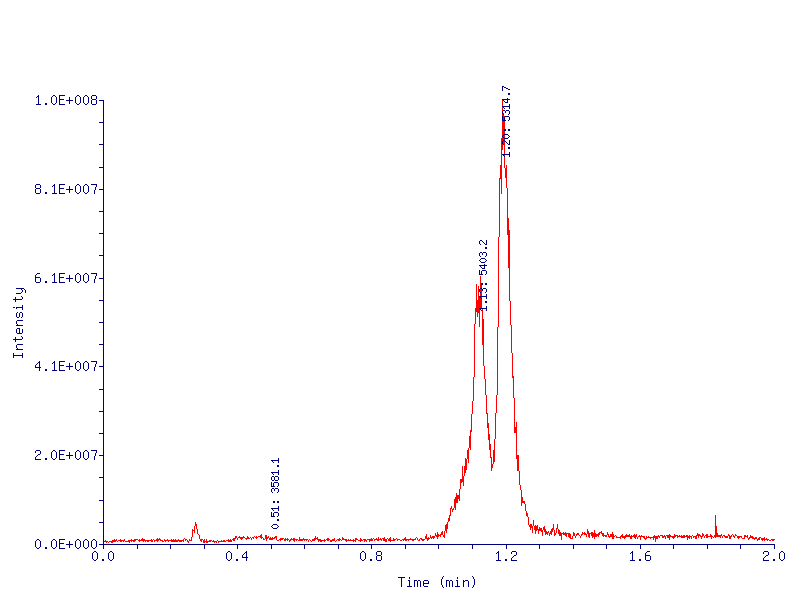


**Figure S57**. LC-MS spectrum of **3t,** expected Mass: 5402; observed Mass: 5403.2


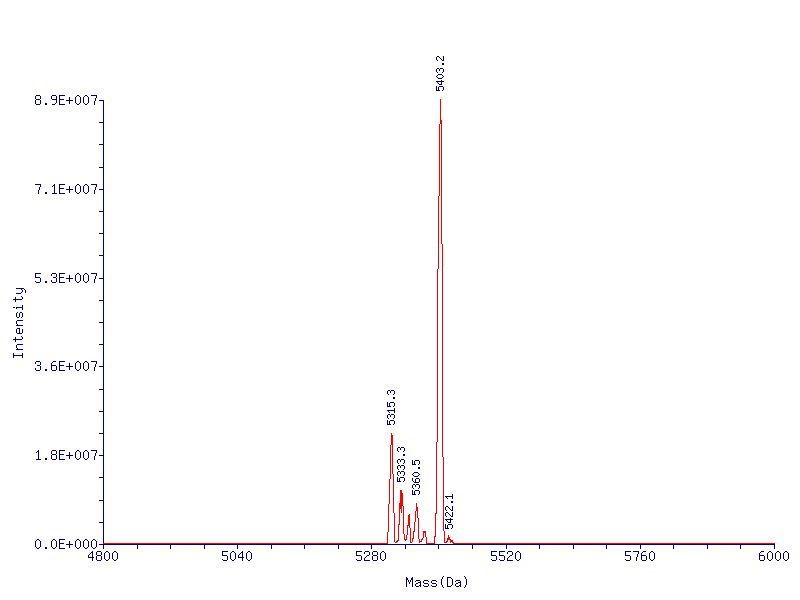


**Figure S58**. Deconvoluted mass spectrum of **3t,** expected Mass: 5402; observed Mass: 5403.2


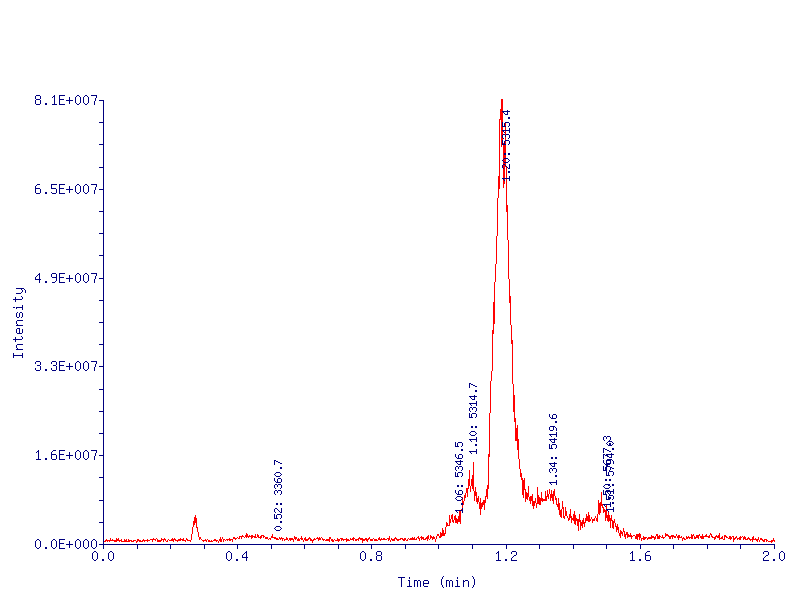


**Figure S59**. LC-MS spectrum of **3u,** expected Mass: 5428; observed Mass: 5428.1


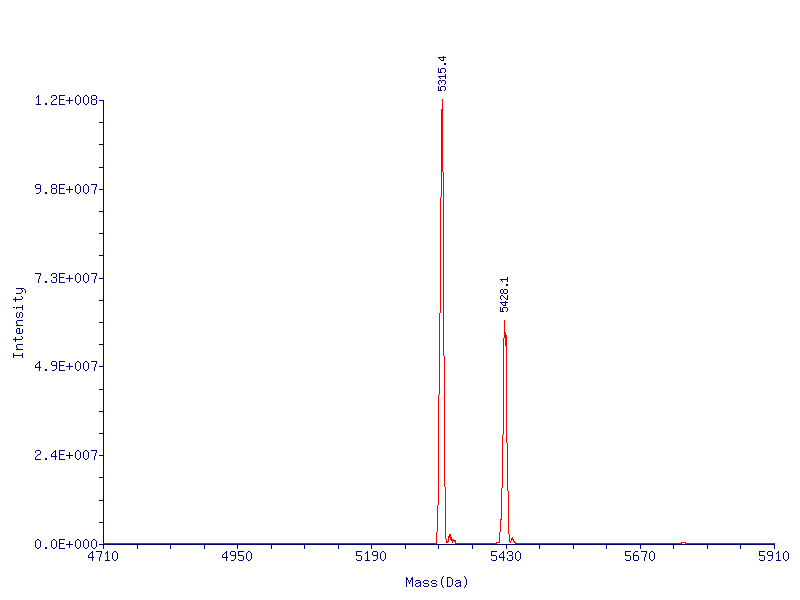


**Figure S60**. Deconvoluted mass spectrum of **3u,** expected Mass: 5428; observed Mass: 5428.1


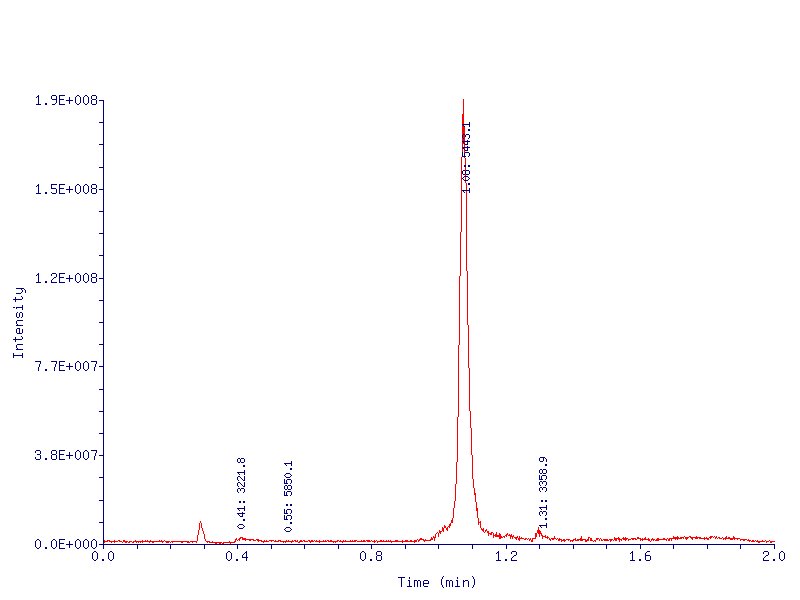


**Figure S61**. LC-MS spectrum of **3w,** expected Mass: 5444; observed Mass: 5443.1


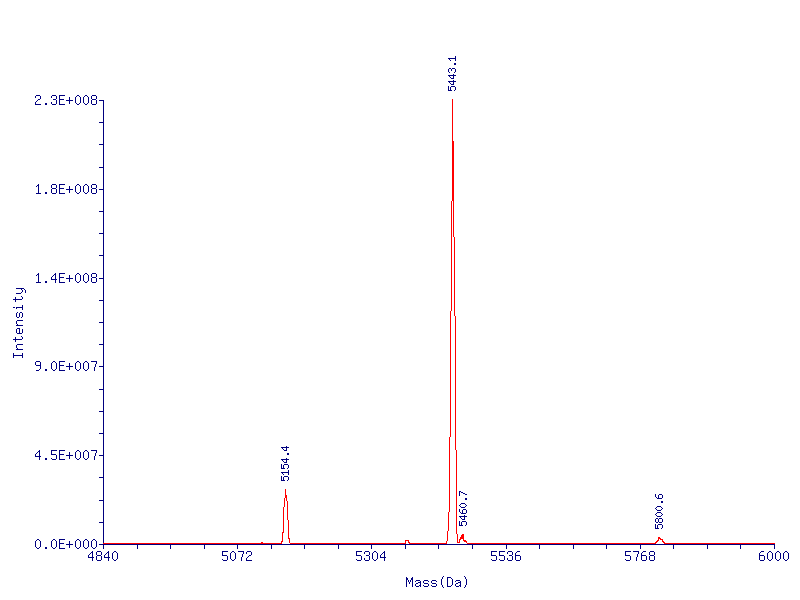


**Figure S62**. Deconvoluted mass spectrum of **3w,** expected Mass: 5444; observed Mass: 5443.1


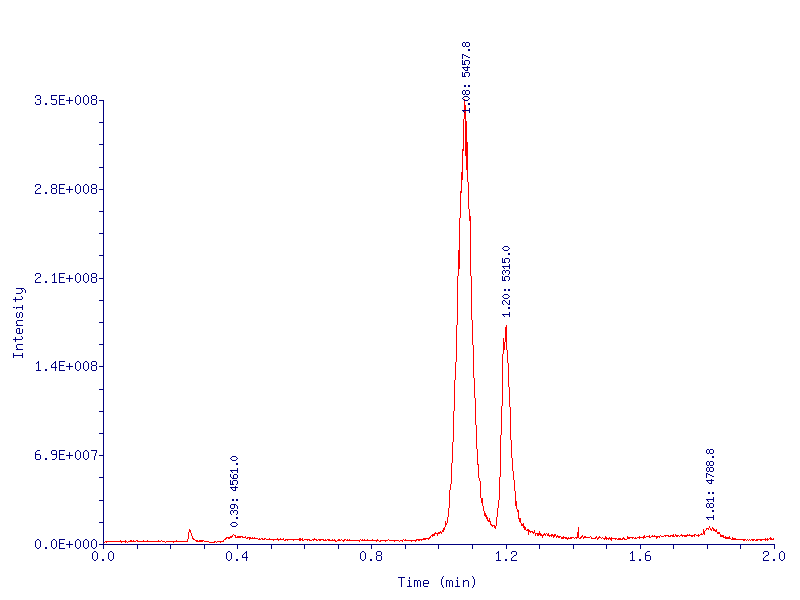


**Figure S63**. LC-MS spectrum of **3x,** expected Mass: 5458; observed Mass: 5457.8


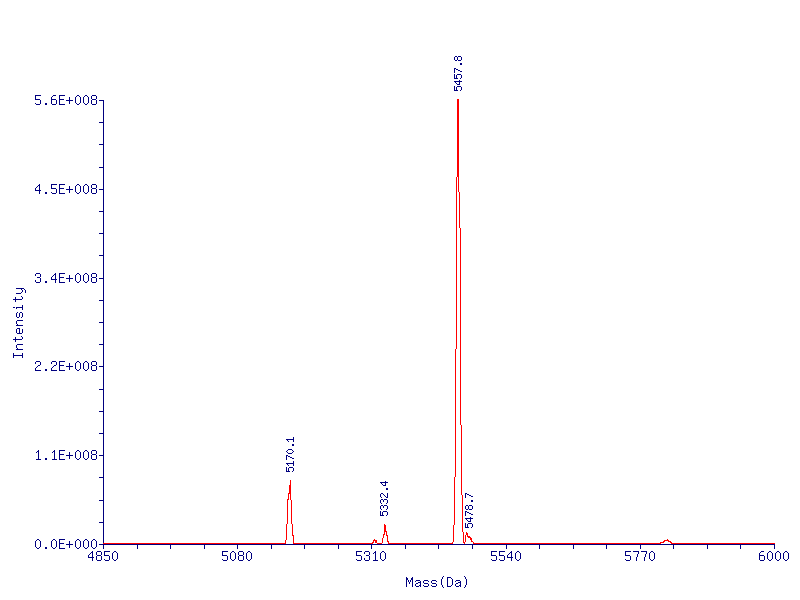


**Figure S64**. Deconvoluted mass spectrum of **3x,** expected Mass: 5458; observed Mass: 5457.8


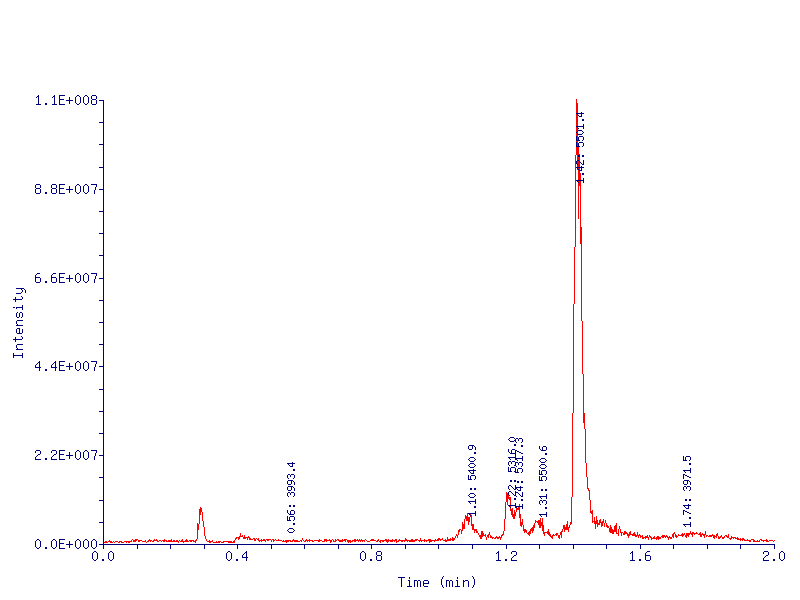


**Figure S65**. LC-MS spectrum of **3y,** expected Mass: 5501; observed Mass: 5501.4


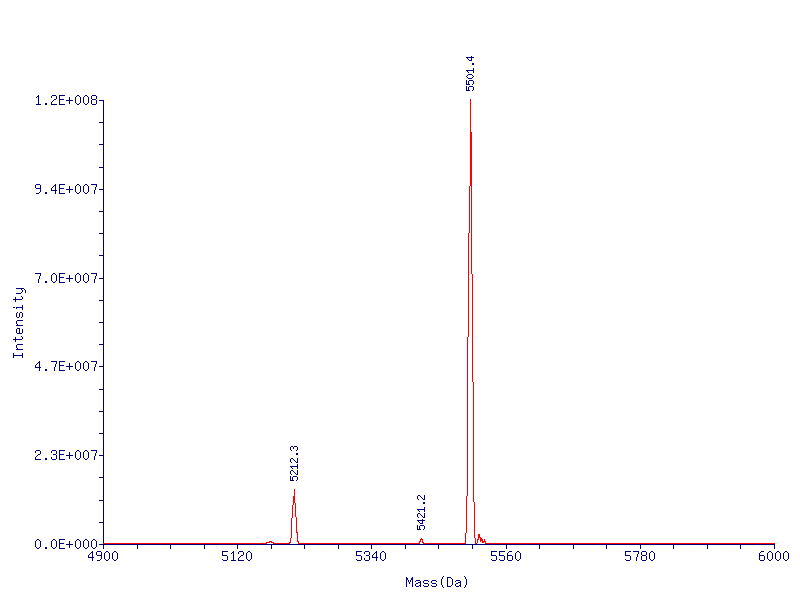


**Figure S66**. Deconvoluted mass spectrum of **3y,** expected Mass: 5501; observed Mass: 5501.4


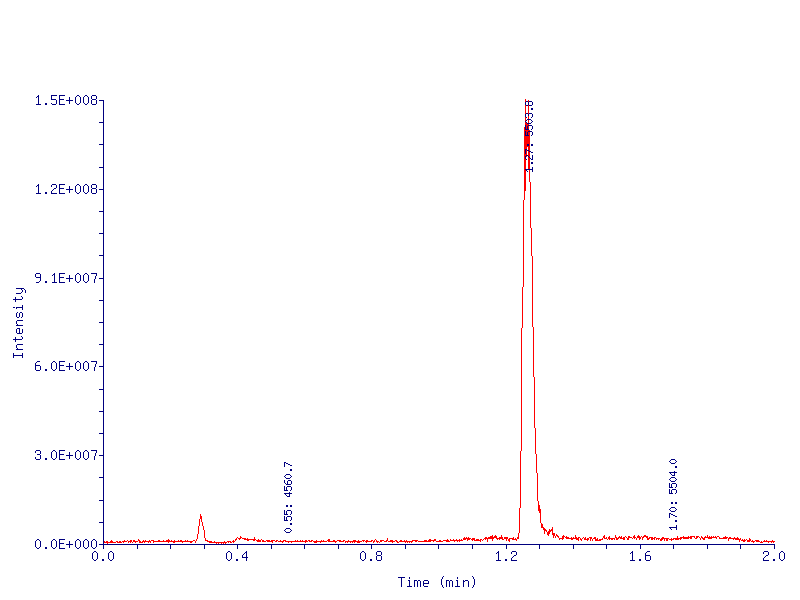


**Figure S67**. LC-MS spectrum of **3z,** expected Mass: 5503; observed Mass: 5503.8


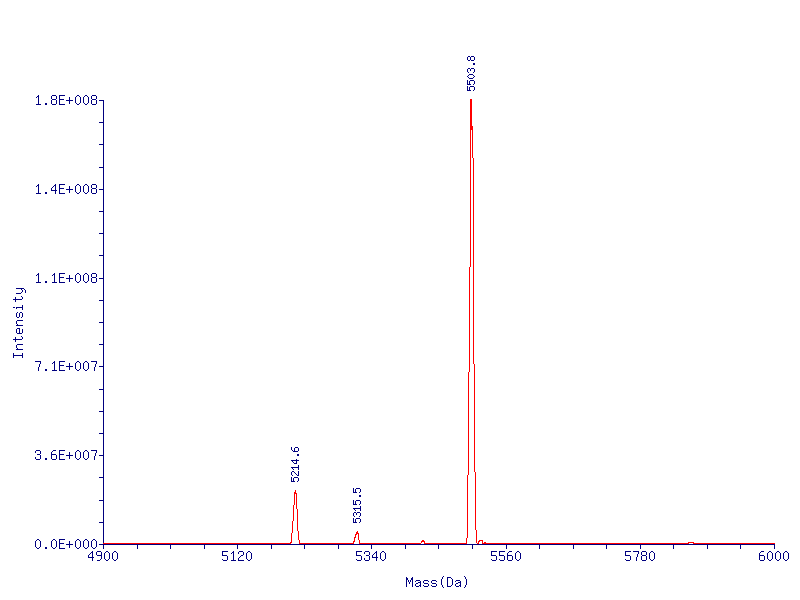


**Figure S68**. Deconvoluted mass spectrum of **3z,** expected Mass: 5503; observed Mass: 5503.8


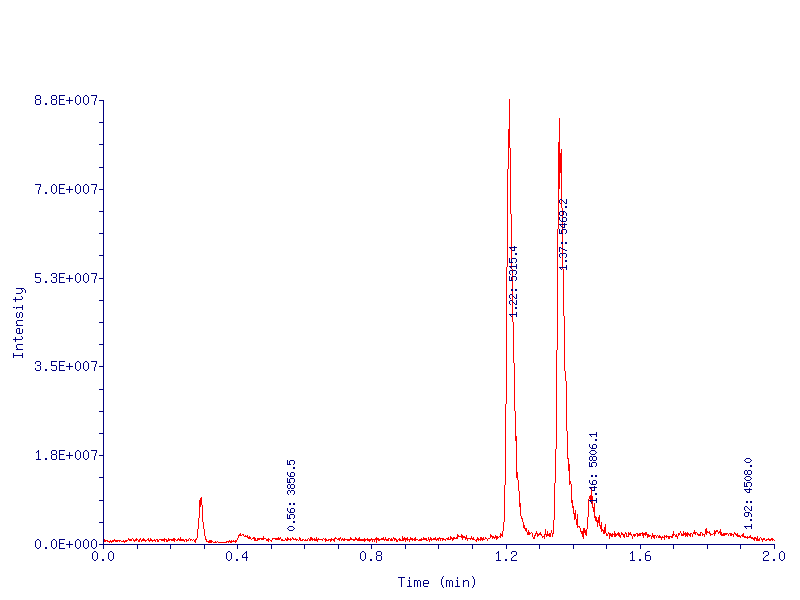


**Figure S69**. LC-MS spectrum of **3aa,** expected Mass: 5468; observed Mass: 5469.2


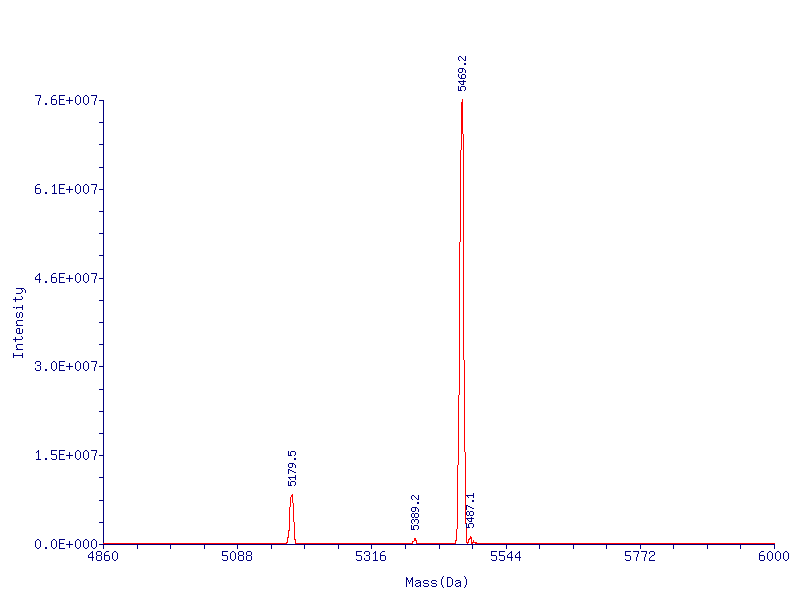


**Figure S70**. Deconvoluted mass spectrum of **3aa,** expected Mass: 5468; observed Mass: 5469.2


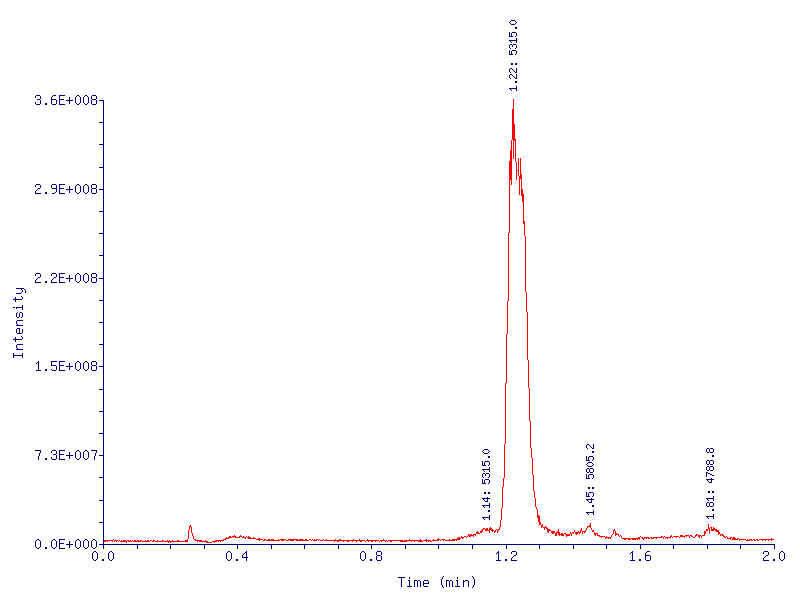


**Figure S71**. LC-MS spectrum of **3ab,** expected Mass: 5515; observed Mass: 5515.5


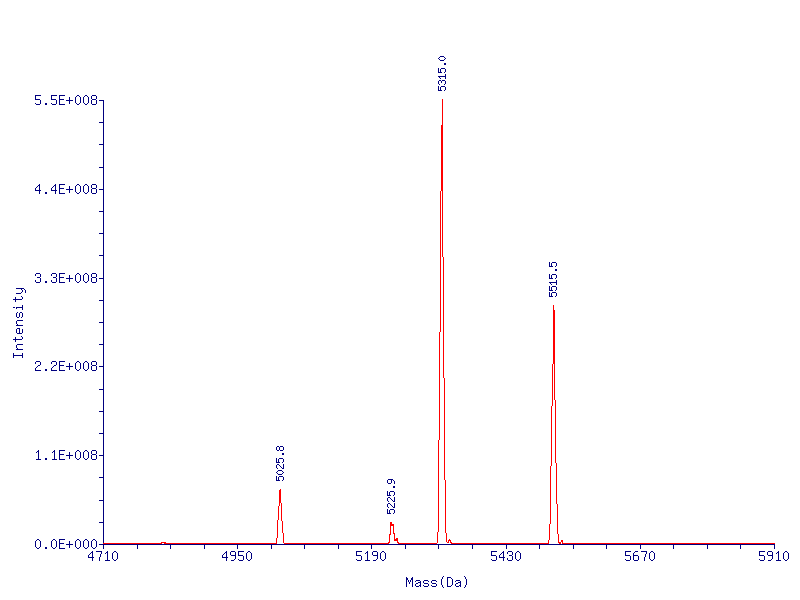


**Figure S72**. Deconvoluted mass spectrum of **3ab,** expected Mass: 5515; observed Mass: 5515.5


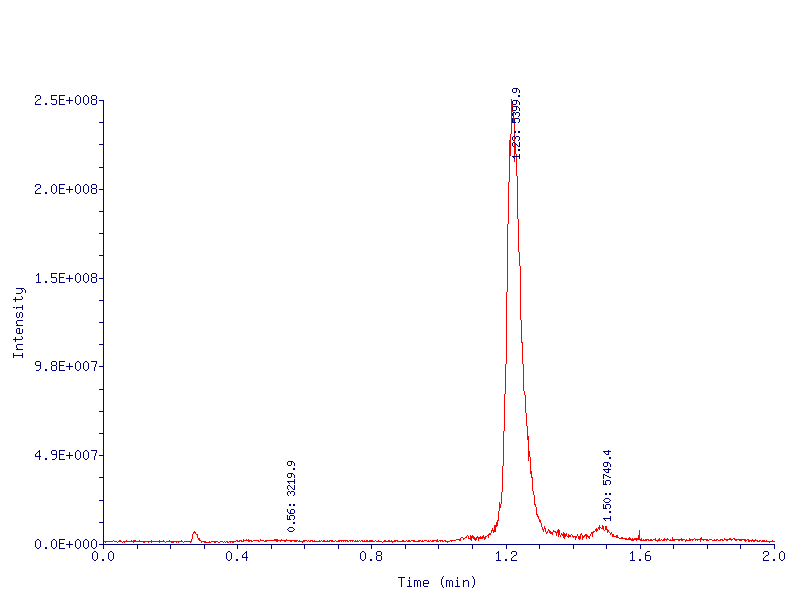


**Figure S73**. LC-MS spectrum of **3ag,** expected Mass: 5401; observed Mass: 5399.9


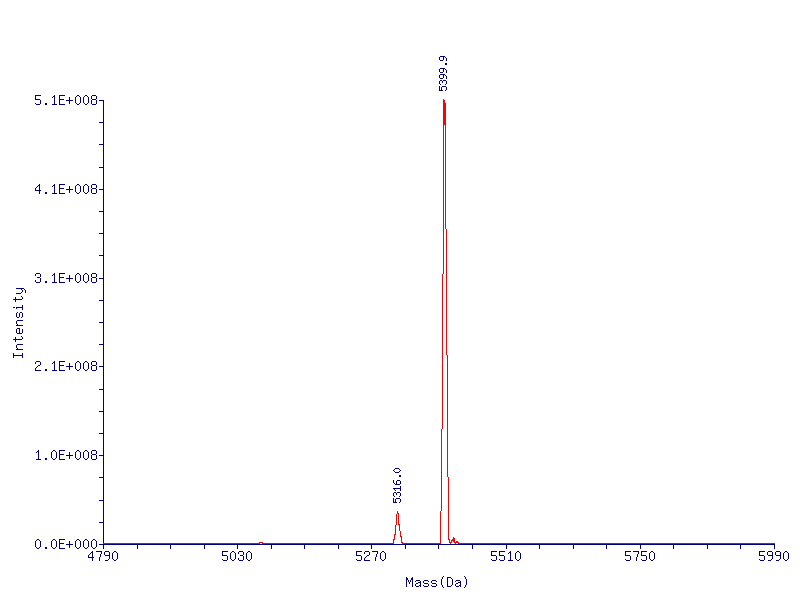


**Figure S74**. Deconvoluted mass spectrum of **3ag,** expected Mass: 5401; observed Mass: 5399.9


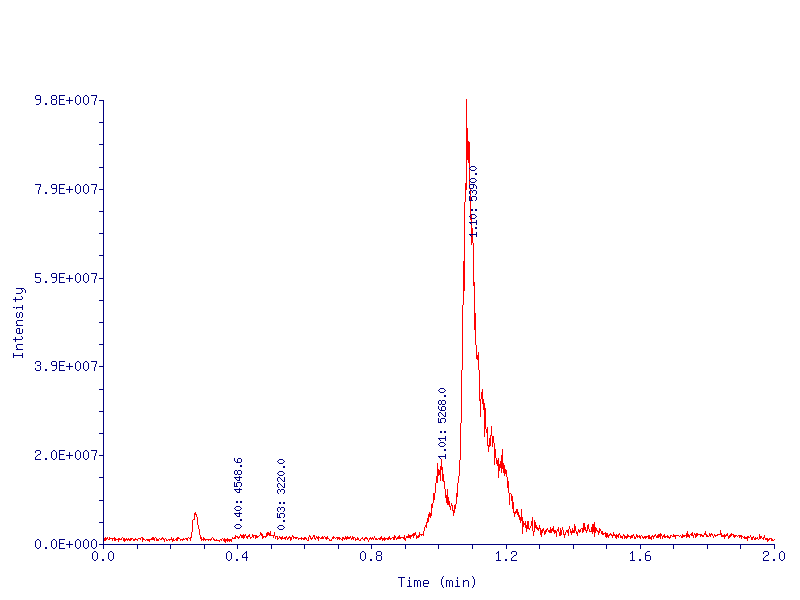


**Figure S75**. LC-MS spectrum of **3ah,** expected Mass: 5390; observed Mass: 5390


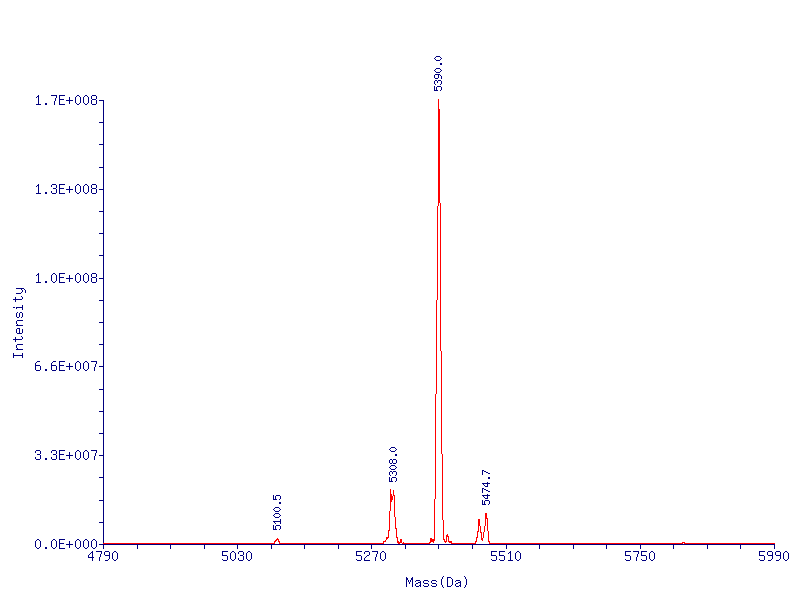


**Figure S76**. Deconvoluted mass spectrum of **3ah,** expected Mass: 5390; observed Mass: 5390


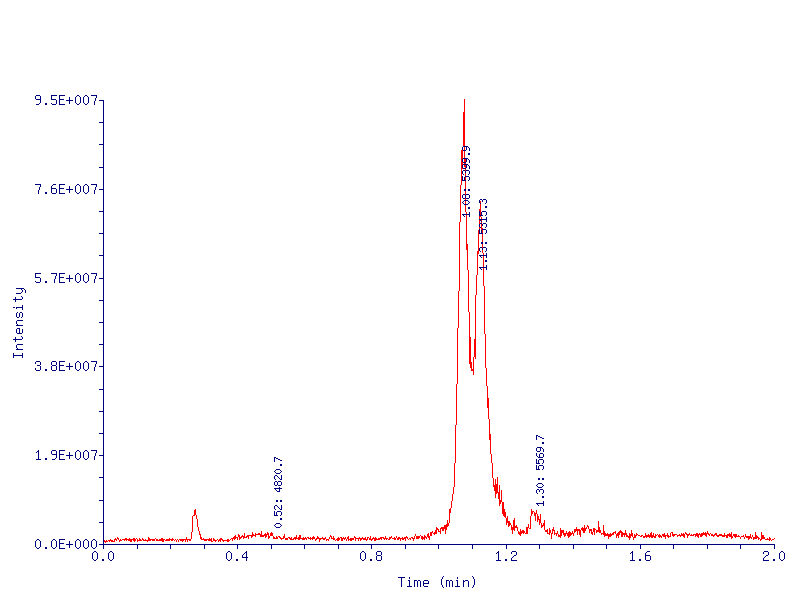


**Figure S77**. LC-MS spectrum of **3ai,** expected Mass: 5401; observed Mass: 5399.9


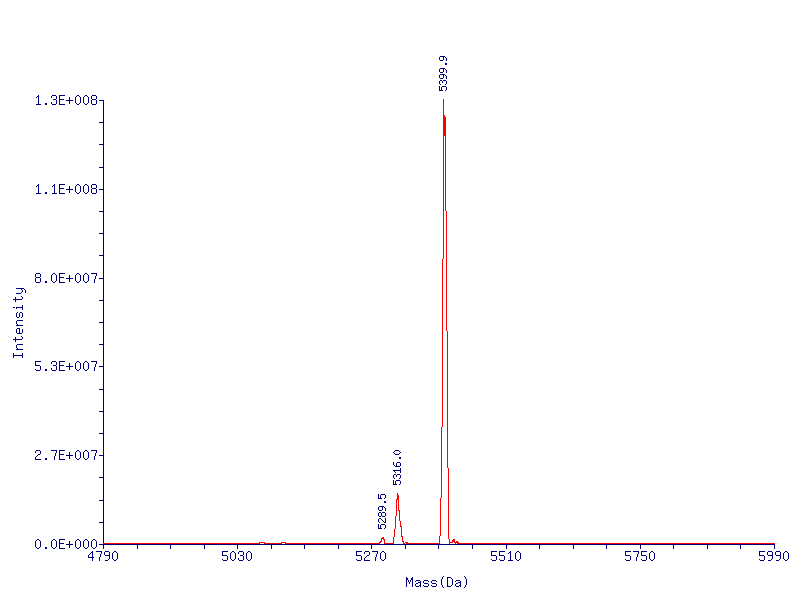


**Figure S78**. Deconvoluted mass spectrum of **3ai,** expected Mass: 5401; observed Mass: 5399.9


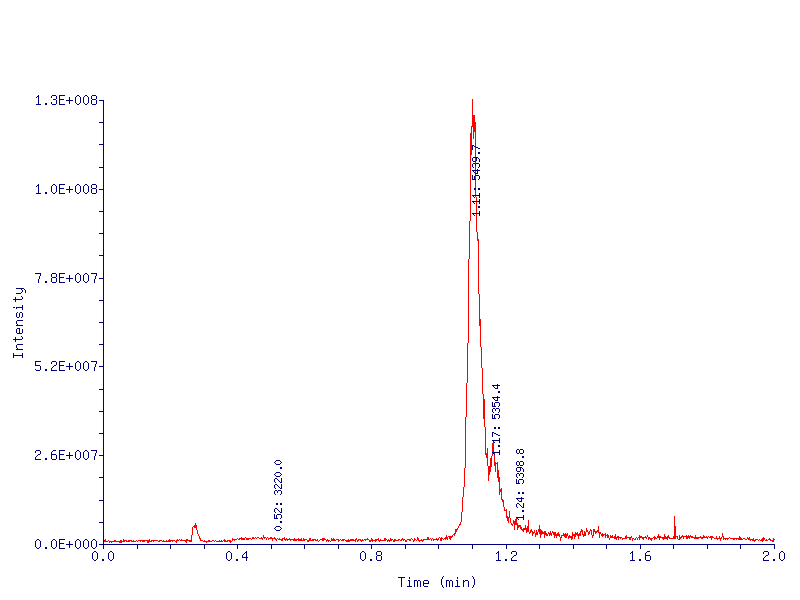


**Figure S79.** LC-MS spectrum of **3aj,** expected Mass: 5440; observed Mass: 5439.7


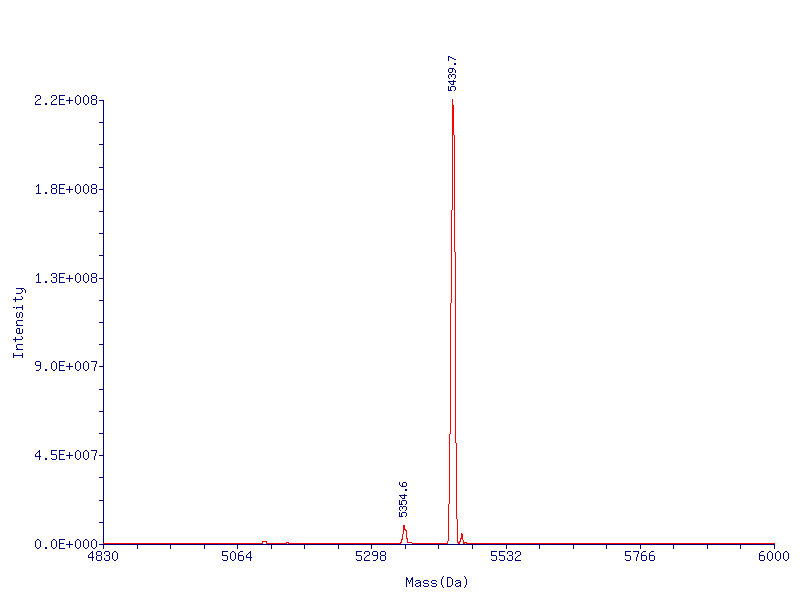


**Figure S80**. Deconvoluted mass spectrum of **3aj,** expected Mass: 5440; observed Mass: 5439.7


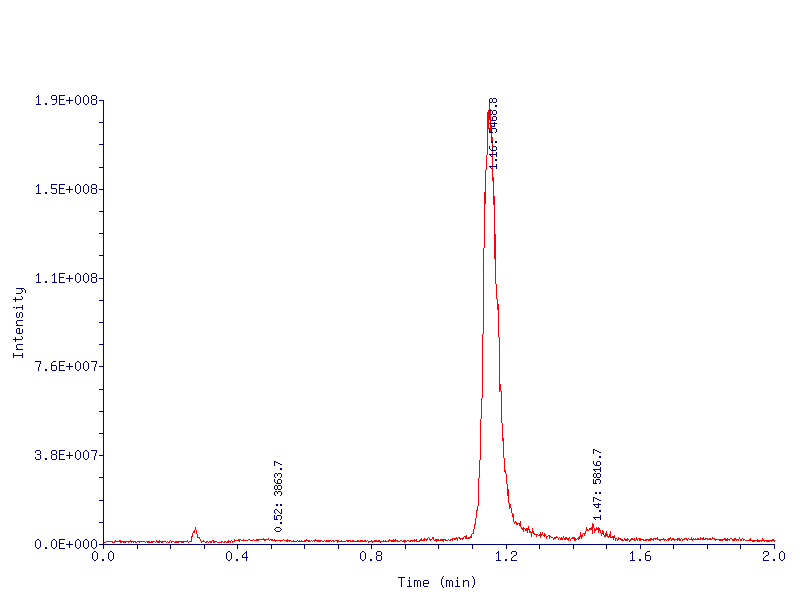


**Figure S81**. LC-MS spectrum of **3ak,** expected Mass: 5468; observed Mass: 5468.8


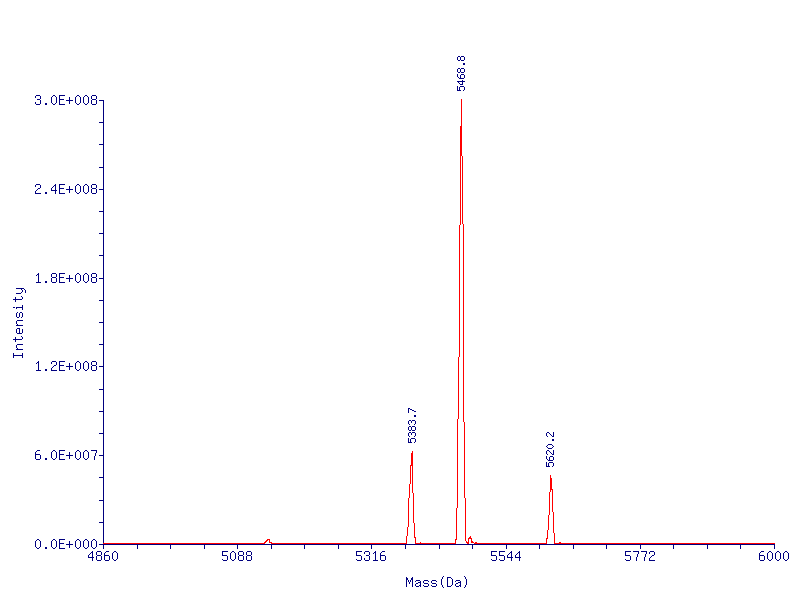


**Figure S82**. Deconvoluted mass spectrum of **3ak,** expected Mass: 5468; observed Mass: 5468.8


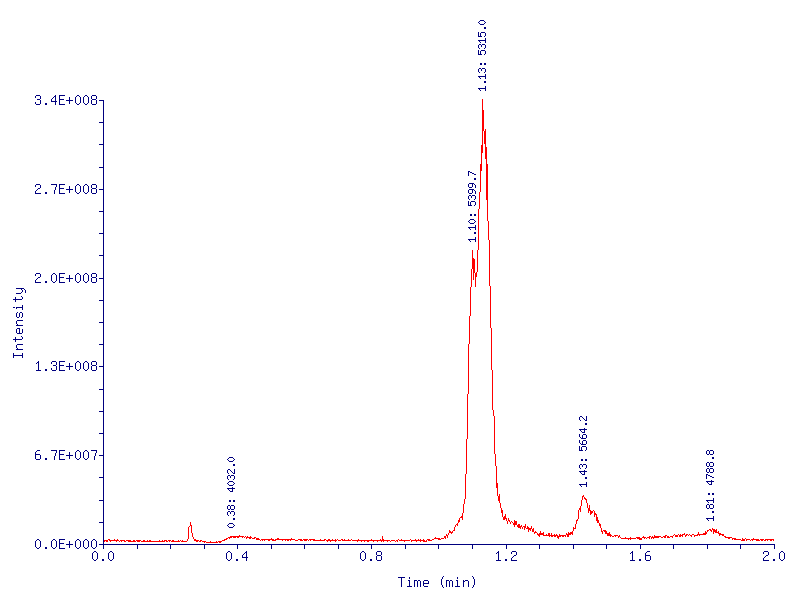


**Figure S83**. LC-MS spectrum of **3al,** expected Mass: 5400; observed Mass: 5399.7


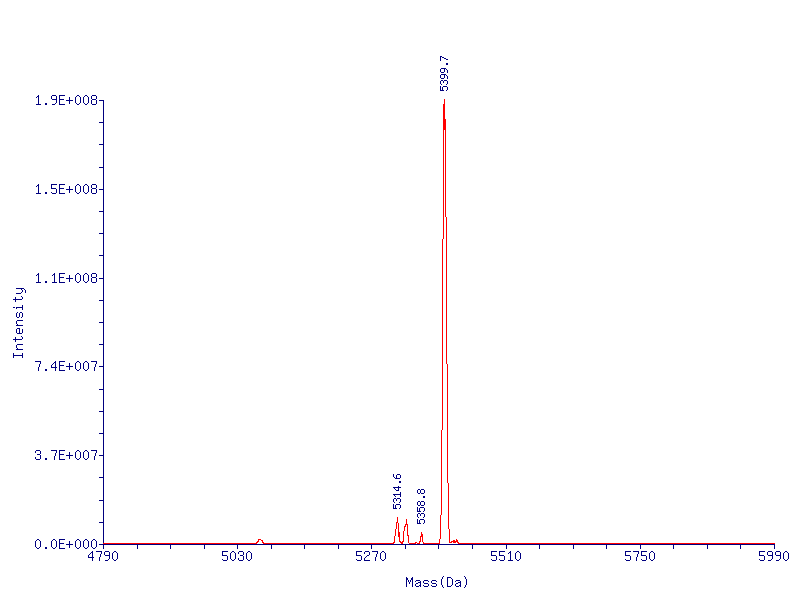


**Figure S84**. Deconvoluted mass spectrum of **3al,** expected Mass: 5400; observed Mass: 5399.7


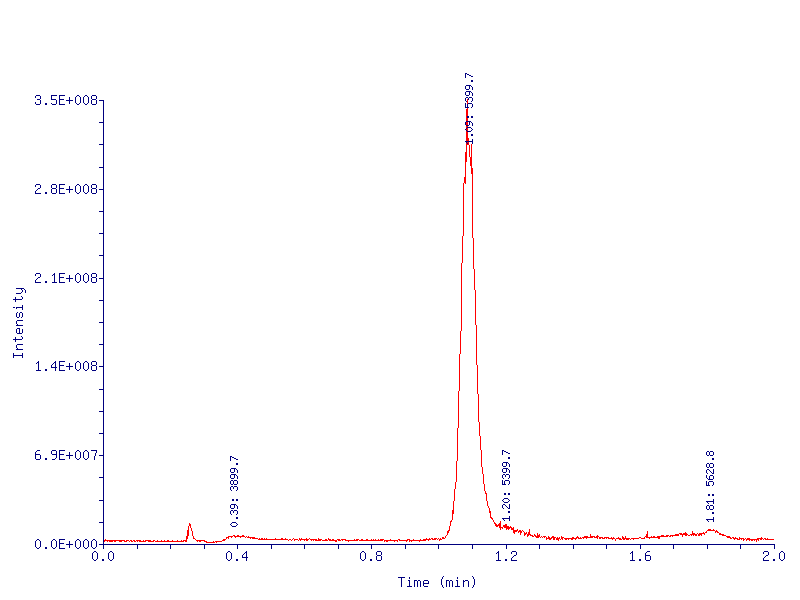


**Figure S85**. LC-MS spectrum of **3am,** expected Mass: 5400; observed Mass: 5399.7


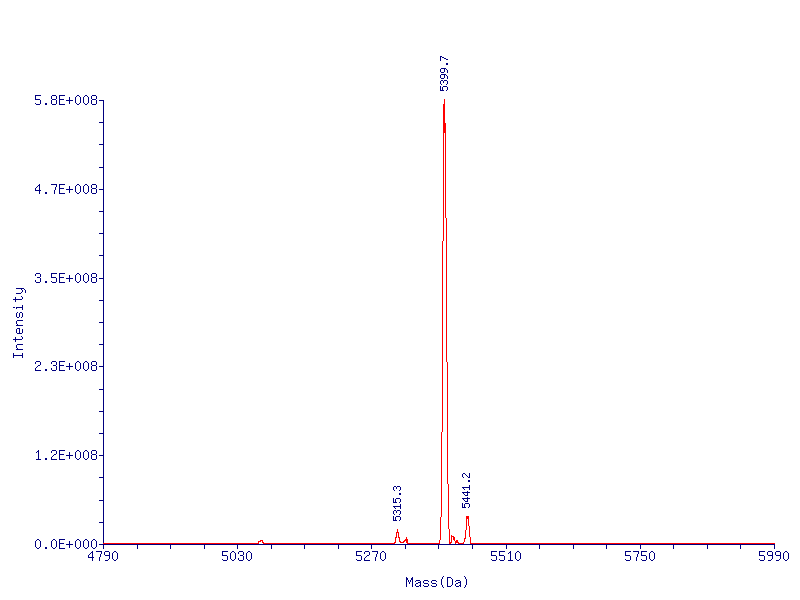


**Figure S86**. Deconvoluted mass spectrum of **3am,** expected Mass: 5400; observed Mass: 5399.7


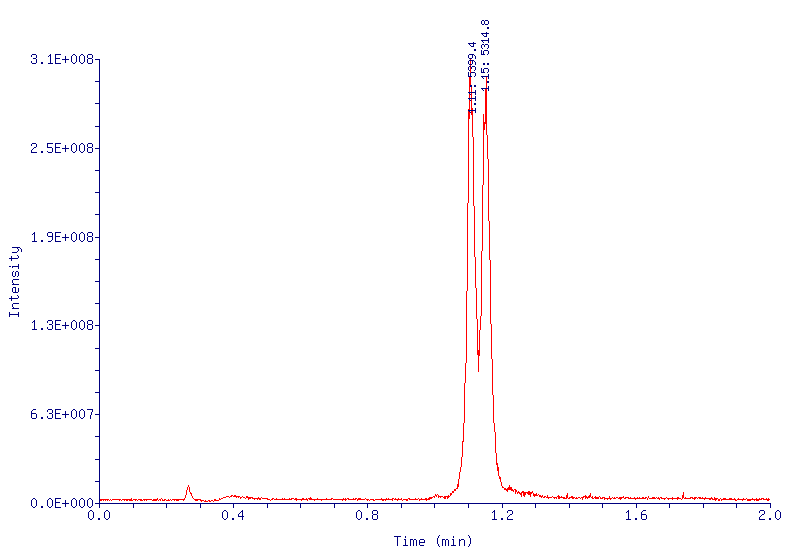


**Figure S87**. LC-MS spectrum of **3an,** expected Mass: 5400; observed Mass: 5399.4


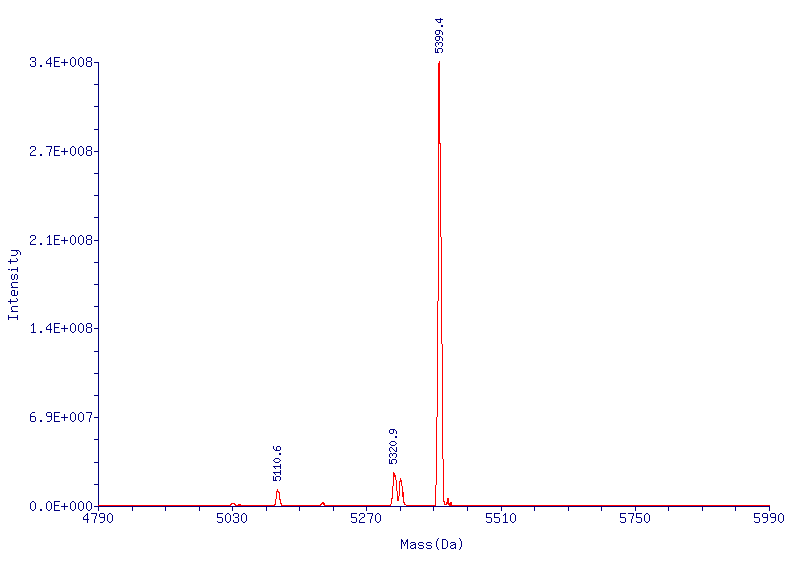


**Figure S88**. Deconvoluted mass spectrum of **3an,** expected Mass: 5400; observed Mass: 5399.4


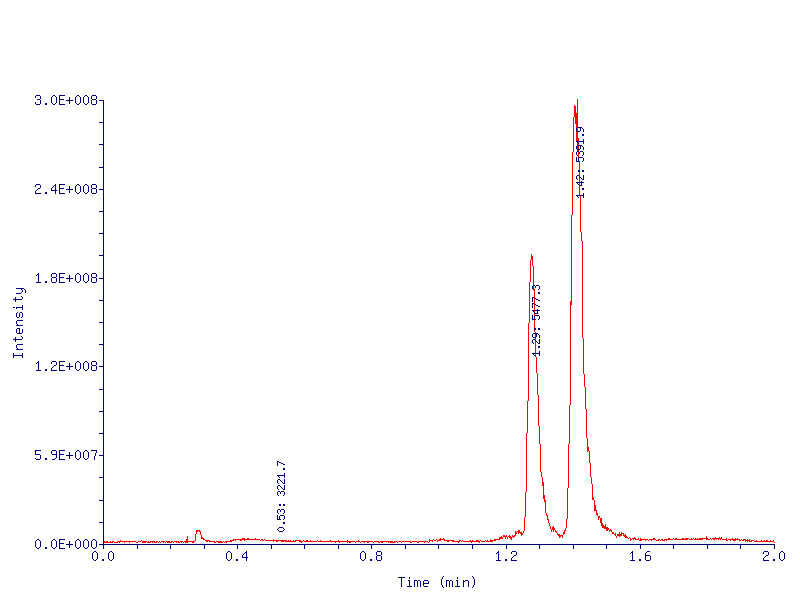


**Figure S89**. LC-MS spectrum of **3ao,** expected Mass: 5477; observed Mass: 5477.3


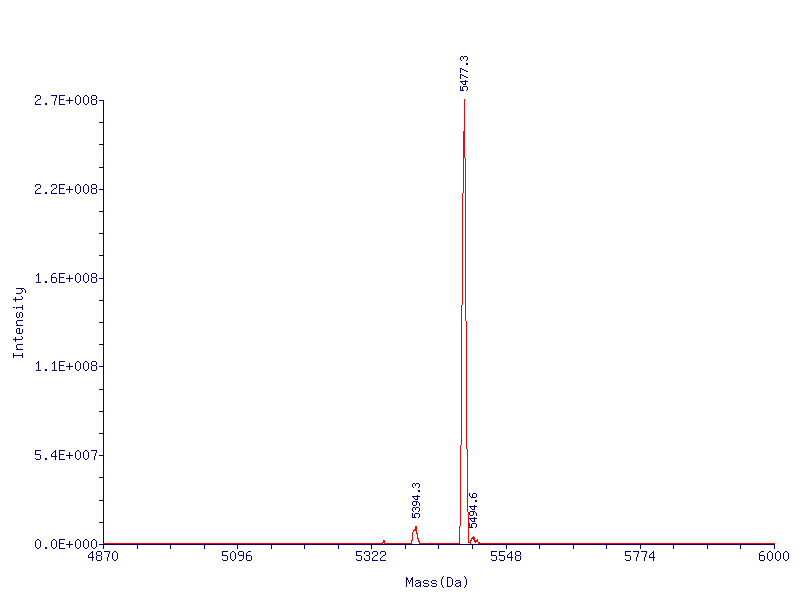


**Figure S90**. Deconvoluted mass spectrum of **3ao,** expected Mass: 5477; observed Mass: 5477.3


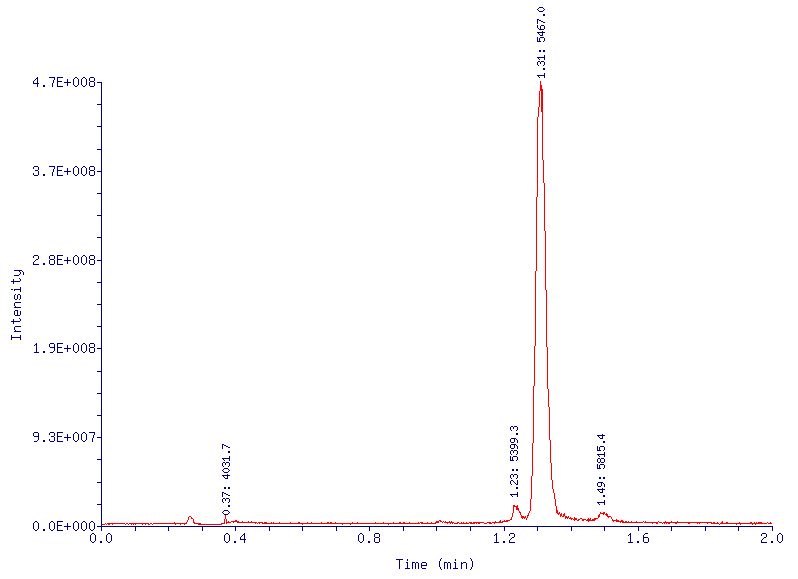


**Figure S91**. LC-MS spectrum of **3ap,** expected Mass: 5467; observed Mass: 5467


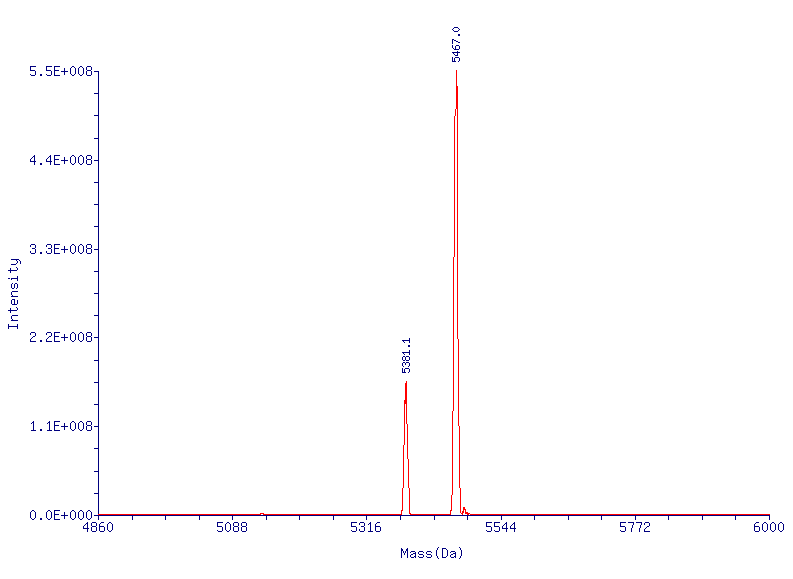


**Figure S92**. Deconvoluted mass spectrum of **3ap,** expected Mass: 5467; observed Mass: 5467


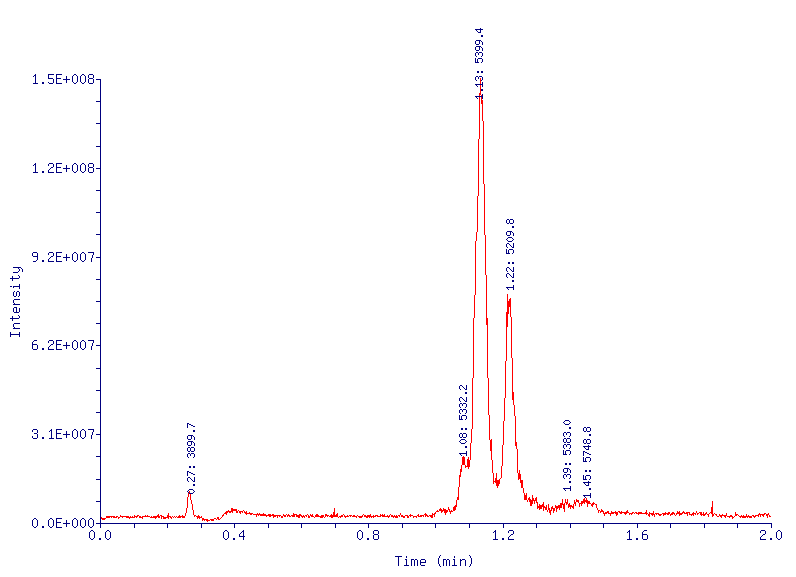


**Figure S93**. LC-MS spectrum of **3aq,** expected Mass: 5400; observed Mass: 5399.4


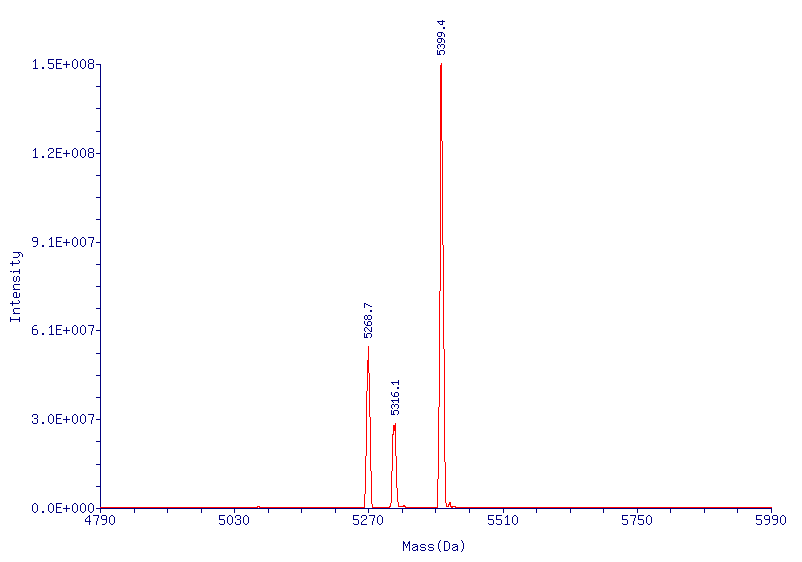


**Figure S94**. Deconvoluted mass spectrum of **3aq,** expected Mass: 5400; observed Mass: 5399.4


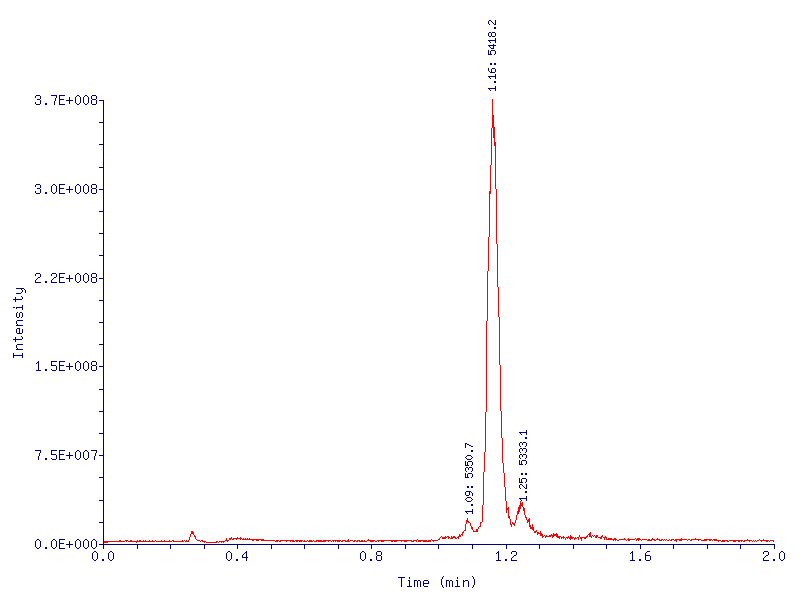


**Figure S95**. LC-MS spectrum of **3ar,** expected Mass: 5418; observed Mass: 5418.2


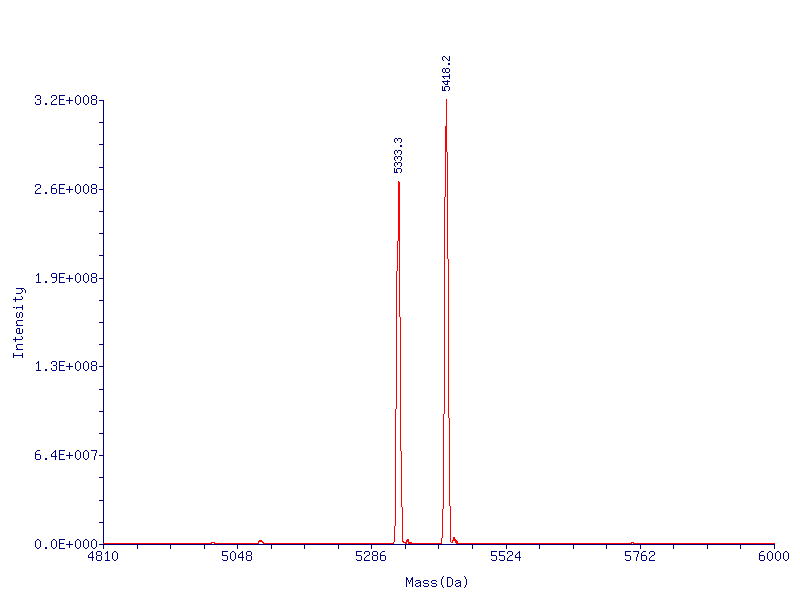


**Figure S96**. Deconvoluted mass spectrum of **3ar,** expected Mass: 5418; observed Mass: 5418.2


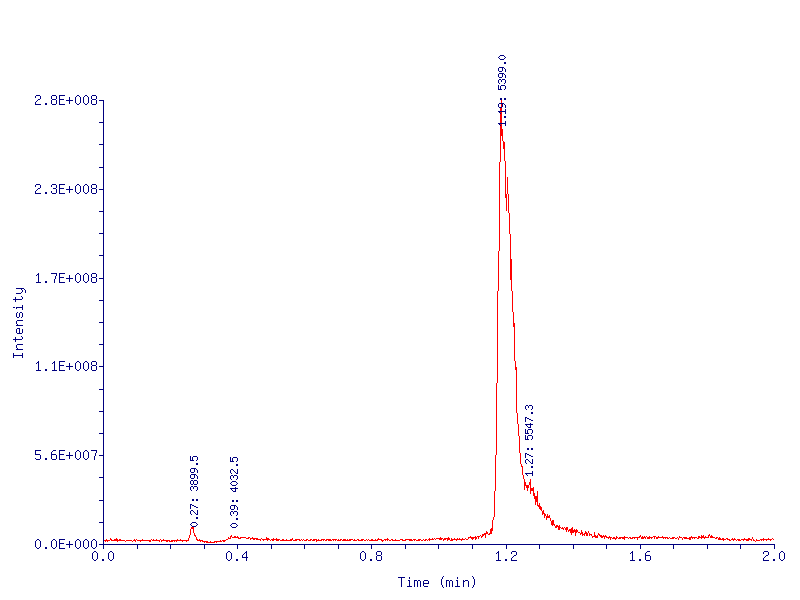


**Figure S97**. LC-MS spectrum of **3as,** expected Mass: 5399; observed Mass: 5399


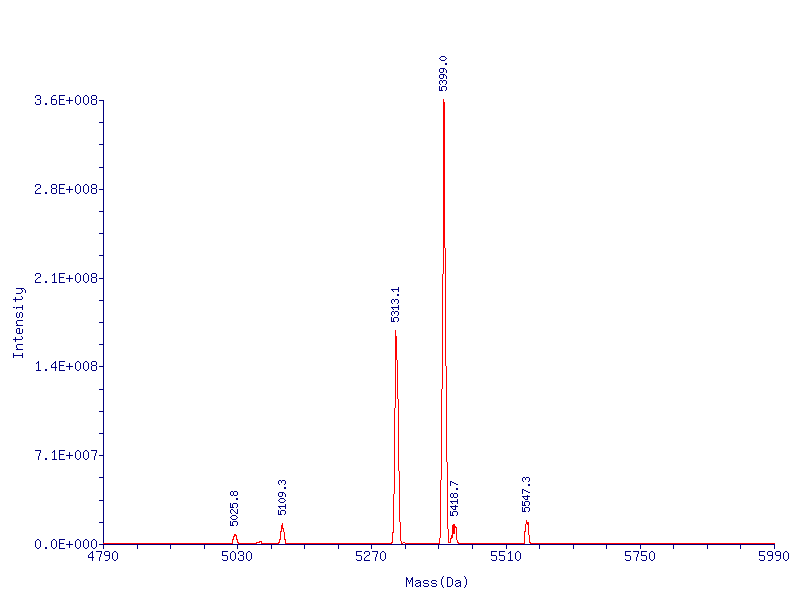


**Figure S98**. Deconvoluted mass spectrum of **3as,** expected Mass: 5399; observed Mass: 5399


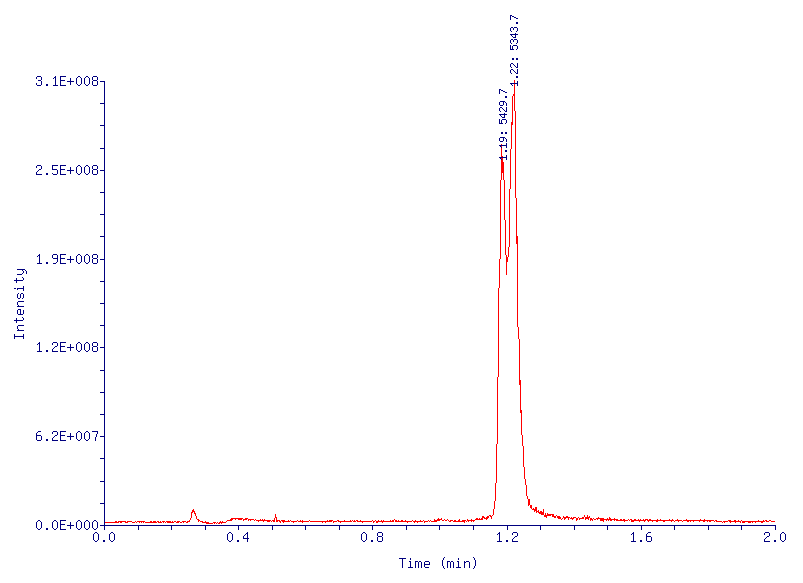


**Figure S99**. LC-MS spectrum of **3at,** expected Mass: 5429; observed Mass: 5429.7


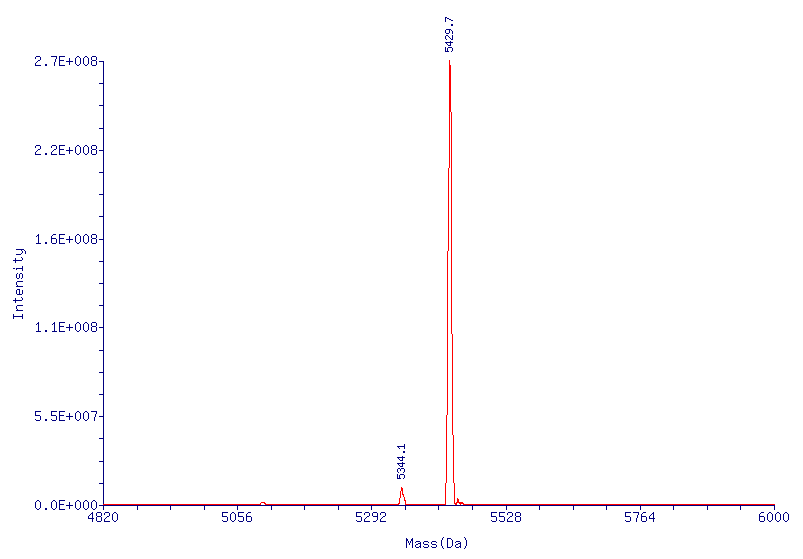


**Figure S100.** Deconvoluted mass spectrum of **3at,** expected Mass: 5429; observed Mass: 5429.7


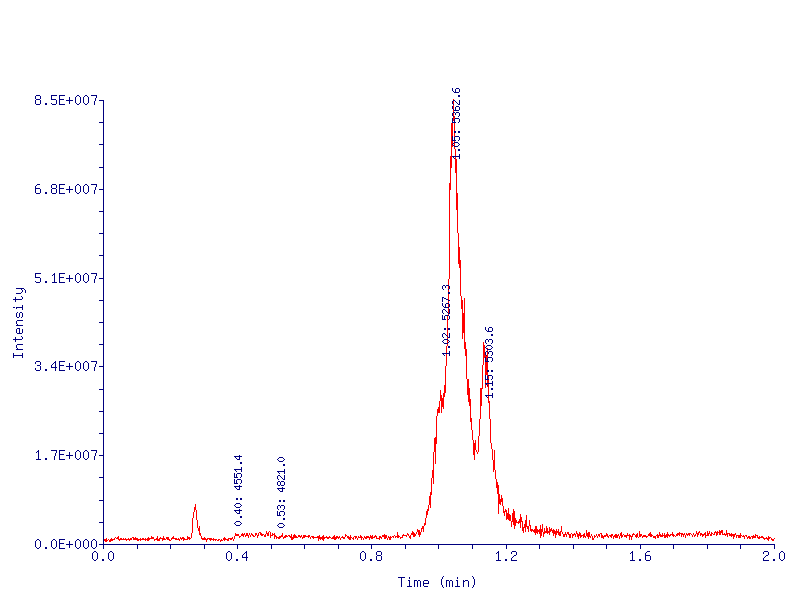


**Figure S101**. LC-MS spectrum of **3au,** expected Mass: 5364; observed Mass: 5362.6


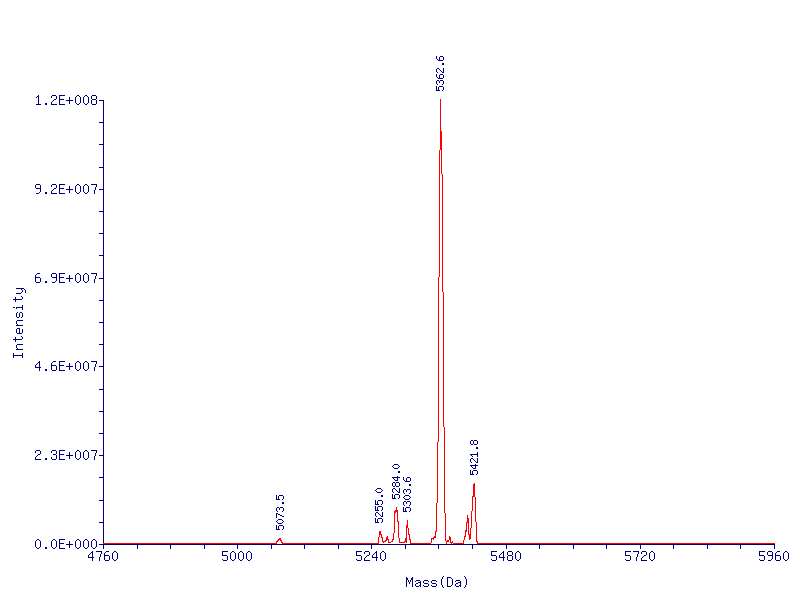


**Figure S102**. Deconvoluted mass spectrum of **3au,** expected Mass: 5364; observed Mass: 5362.6


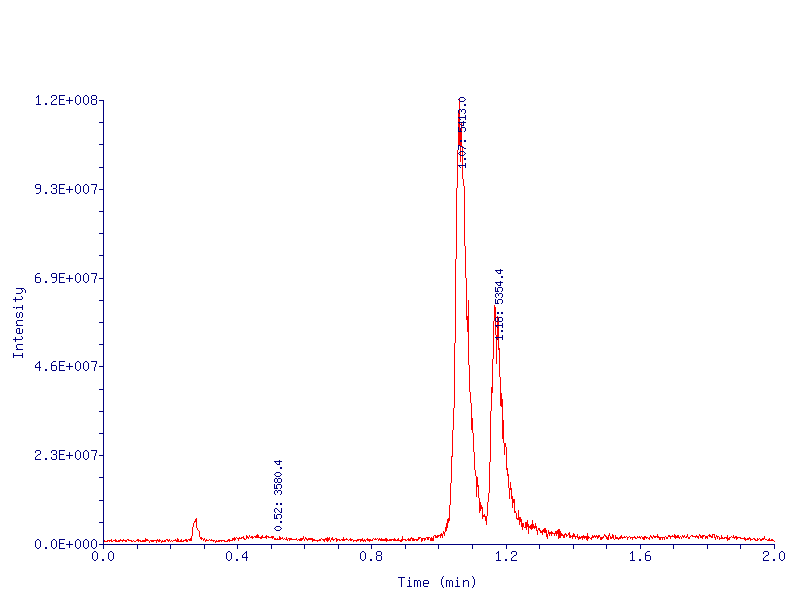


**Figure S103**. LC-MS spectrum of **3av,** expected Mass: 5414; observed Mass: 5413

**Figure S104**. Deconvoluted mass spectrum of **3av,** expected Mass: 5414; observed Mass: 5413

**Figure S105**. LC-MS spectrum of **3aw,** expected Mass: 5442; observed Mass: 5442.3

**Figure S106**. Deconvoluted mass spectrum of **3aw,** expected Mass: 5442; observed Mass: 5442.3

**Figure S107**. LC-MS spectrum of **3ax,** expected Mass: 5476; observed Mass: 5475.5

**Figure S108**. Deconvoluted mass spectrum of **3ax,** expected Mass: 5476; observed Mass: 5475.5

**Figure S109**. LC-MS spectrum of **3ay,** expected Mass: 5454; observed Mass: 5453.5

**Figure S110**. Deconvoluted mass spectrum of **3ay,** expected Mass: 5454; observed Mass: 5453.5

**Figure S111**. LC-MS spectrum of **3az,** expected Mass: 5512; observed Mass: 5512.1

**Figure S112**. Deconvoluted mass spectrum of **3az,** expected Mass: 5512; observed Mass: 5512.1

**Figure S113**. LC-MS spectrum of **3ba,** expected Mass: 5472; observed Mass: 5473.3

**Figure S114**. Deconvoluted mass spectrum of **3ba,** expected Mass: 5472; observed Mass: 5473.3

**Figure S115**. LC-MS spectrum of **3bb,** expected Mass: 5522; observed Mass: 5522.2

**Figure S116**. Deconvoluted mass spectrum of **3bb,** expected Mass: 5522; observed Mass: 5522.2

**Figure S117**. LC-MS spectrum of **3bc,** expected Mass: 5455; observed Mass: 5455

**Figure S118**. Deconvoluted mass spectrum of **3bc,** expected Mass: 5455; observed Mass: 5455

**Figure S119**. LC-MS spectrum of **3bd,** expected Mass: 5392; observed Mass: 5391.4

**Figure S120**. Deconvoluted mass spectrum of **3bd,** expected Mass: 5392; observed Mass: 5391.4

**Figure S121**. LC-MS spectrum of **3be,** expected Mass: 5403; observed Mass: 5402.6

**Figure S122**. Deconvoluted mass spectrum of **3be,** expected Mass: 5403; observed Mass: 5402.6

**Figure S123**. LC-MS spectrum of **3bf,** expected Mass: 5442; observed Mass: 5442.2

**Figure S124**. Deconvoluted mass spectrum of **3bf,** expected Mass: 5442; observed Mass: 5442.2

**Figure S125**. LC-MS spectrum of **3bg,** expected Mass: 5468; observed Mass: 5468.7

**Figure S126**. Deconvoluted mass spectrum of **3bg,** expected Mass: 5468; observed Mass: 5468.7

**Figure S127**. LC-MS spectrum of **3bh,** expected Mass: 5429; observed Mass: 5428.3

**Figure S128**. Deconvoluted mass spectrum of **3bh,** expected Mass: 5429; observed Mass: 5428.3

**Figure S129**. LC-MS spectrum of **3bi,** expected Mass: 5418; observed Mass: 5417.3

**Figure S130**. Deconvoluted mass spectrum of **3bi,** expected Mass: 5418; observed Mass: 5417.3
